# Structural and enzymatic plasticity of SIRT6 deacylase activity

**DOI:** 10.1016/j.jbc.2025.108446

**Published:** 2025-03-25

**Authors:** Zhipeng A. Wang, Jonathan Markert, Samuel D. Whedon, Maheeshi Yapa Abeywardana, Xinlei Sheng, Eunju Nam, Kwangwoon Lee, Maggie Chen, Amanda Waterbury, Yingming Zhao, Lucas Farnung, Philip A. Cole

**Affiliations:** 1 Division of Genetics, Department of Medicine, Brigham and Women's Hospital, Boston, Massachusetts, United States; 2 Department of Biological Chemistry and Molecular Pharmacology, Harvard Medical School, Boston, Massachusetts, United States; 3 Desai Sethi Urology Institute & Sylvester Comprehensive Cancer Center, University of Miami Miller School of Medicine, Miami, Florida, United States; 4 Department of Cell Biology, Blavatnik Institute, Harvard Medical School, Boston, Massachusetts, United States; 5 Ben May Department of Cancer Research, The University of Chicago, Chicago, Illinois, USA

**Keywords:** chromatin, chromatin modification, histone deacetylase, histone modification, sirtuin

## Abstract

Sirtuin 6 (SIRT6) is an NAD-dependent protein deacylase that targets lysine residues in histones in the cell nucleus, where it helps maintain genome stability and links metabolism to epigenetic control. Dysregulation of SIRT6 is believed to be associated with aging and cancer, making it of pharmacological interest. In this study, we use cryo-EM and enzymology to explore SIRT6 preference and adaptability toward different nucleosomal substrates. We have visualized a trapped complex of SIRT6 in the process of deacylating H3K27, demonstrating how SIRT6 undergoes conformational changes to remove differently positioned histone marks. Additional biochemical studies further reveal the plasticity of SIRT6, which accommodates various metabolism-linked modifications, such as lysine lactylation and **β**-hydroxybutyrylation. To further understand the basis for substrate selectivity of SIRT6, we explore the effects of an established G60A enzyme mutation, proximal H3 modifications, and small-molecule modulators. These findings highlight the versatility of SIRT6 and provide key mechanistic insights into its molecular recognition.

The nucleosome is the basic building block of chromatin and regulates accessibility of DNA in various biological processes (1). Nucleosomes are comprised of two copies of the core histones H2A, H2B, H3, and H4, which form an octamer that tightly packs DNA (2). These histones are subject to post-translational modifications (PTMs) including lysine acetylation and acylation, which regulate the recruitment of downstream effectors and influence the accessibility of the chromatin (3).

Histone modifications are dynamically regulated by enzymatic erasers such as Sirtuin 6 (SIRT6), an NAD-dependent deacylase (4). SIRT6 has been reported to play roles (5) in maintaining genome stability (6, 7), regulating aging processes (8, 9), DNA damage repair (10, 11), and connecting metabolism with epigenetic regulation (12, 13). Recognized as a potential tumor suppressor (14), SIRT6 is considered a possible therapeutic target (15). Notably, allosteric activation of SIRT6 by small molecules like MDL-800 has shown promise as a potential anticancer treatment (16).

Cellular studies indicate that SIRT6 predominantly deacetylates histone H3 at lysine 9 (H3K9ac) (17) and histone H3 at lysine 18 (18). SIRT6 prefers acetylated histone H3 incorporated into nucleosomes (19), distinguishing it from other sirtuin family members that prefer free histone substrates (20). This specificity is attributed to recognition of the nucleosome acidic patch by its unique zinc-binding loop and interactions with nucleosomal DNA, which position it to selectively deacylate histone H3 (21, 22). In prior work, we visualized SIRT6’s interaction with the nucleosome using an H3K9-methyl-thiourea (MTU)–modified nucleosome that traps the enzyme–substrate complex. While SIRT6 erases H3K9ac fastest among the N-terminal H3 acetylations, it exhibits robust activity against multiple acetylation sites, such as H3K27ac, and longer linear acylations *in vitro* (21, 23). However, exactly how SIRT6 accommodates these different substrates remains unclear. Importantly, H3K27ac is a key epigenetic mark associated with active gene transcription (24) and its dysregulation is linked to various cancers. Therefore, elucidating the mechanism by which SIRT6 deacetylates H3K27 is of particular significance. Furthermore, the regulation of SIRT6 by neighboring PTMs, small molecules, and mutations (25) remains poorly understood, particularly where nucleosome substrates are concerned.

In this study, we probe the plasticity of SIRT6 across a range of dimensions. We use cryo-EM to capture SIRT6 in a state poised to deacetylate H3K27. We investigate the role of Gly60 in SIRT6 flexibility and substrate selectivity and the effects of previously reported small-molecule activators on SIRT6 kinetics. Finally, we explore how local histone H3 PTMs affect SIRT6’s ability to deacetylate its substrates. Together, these insights provide a structural and mechanistic framework for understanding the substrate plasticity of SIRT6.

## Results

### Structural analysis of SIRT6 engaged with an H3K27MTU nucleosome construct

**Figure 1:**
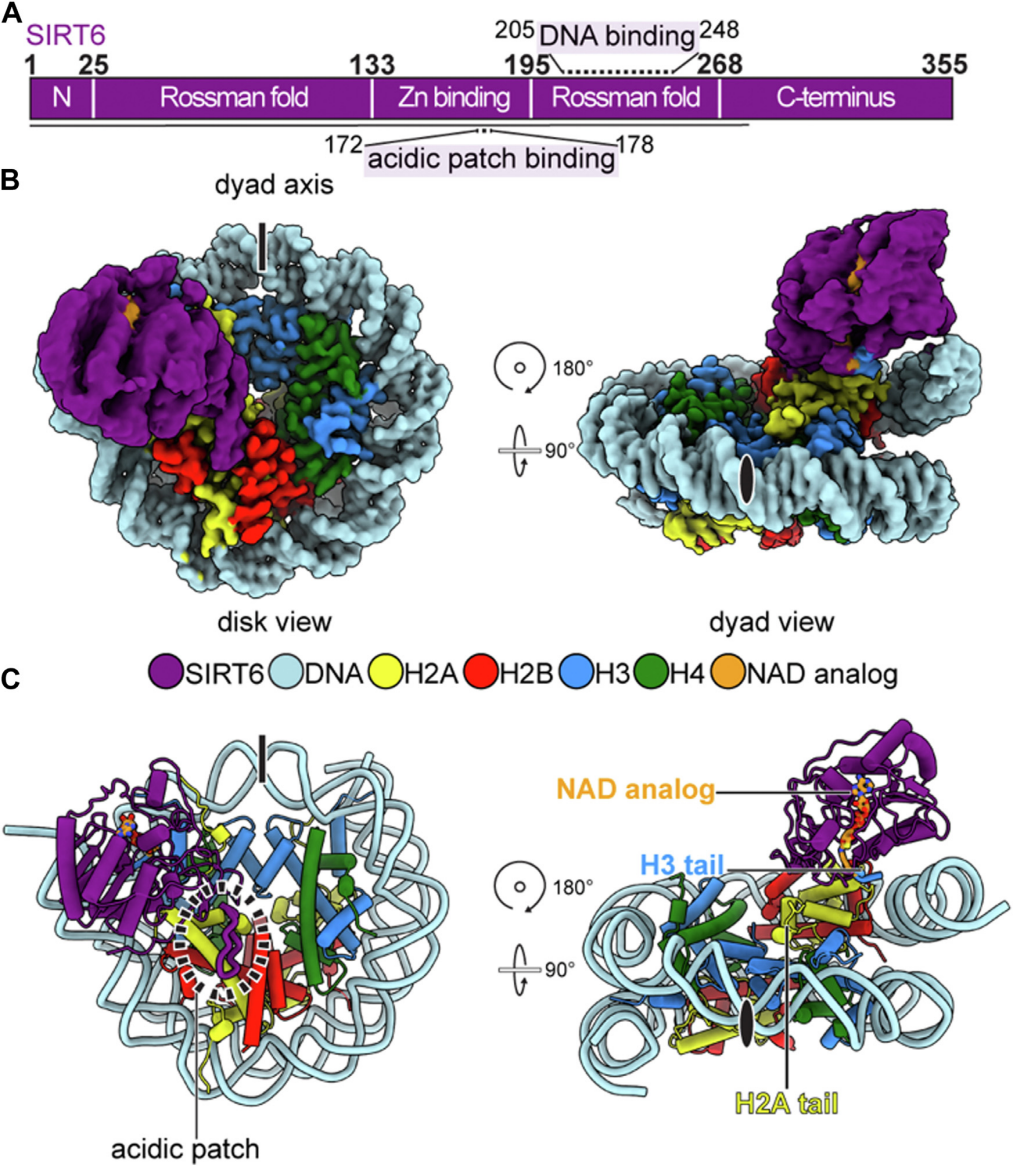
**Cryo-EM structure of SIRT6 trapped with H3K27MTU nucleosome**. *A*, domain map of SIRT6, modeled region is highlighted with a *black line*. *B* and *C*, two views of density map (*B*) and model (*C*) are presented. Colors are indicated in the panel. H3K27, histone H3 at lysine 27; MTU, methyl-thiourea; SIRT6, sirtuin 6.

To understand the mechanisms of plasticity in substrate recognition by SIRT6, we employed cryo-EM to visualize a complex of SIRT6 bound to a H3K27MTU containing nucleosomal substrate. We reconstituted nucleosomes with H3K27MTU and 185 base pairs of DNA. In the presence of NAD, SIRT6 forms a covalent intermediate analog with the MTU group (21) allowing us to capture this normally transient interaction (Fig. S1). Using single-particle cryo-EM, we determined the structure of the SIRT6–H3K27MTU–nucleosome complex at an overall resolution of 3.2 Å (Fourier shell correlation: 0.143) from 124,724 particles with good density for SIRT6 on one side of the nucleosomal disc (Fig. 1). Local resolution for the nucleosome was around 3 Å, whereas SIRT6 ranged from 3 Å (near the acidic patch interaction) to ≥6 Å (near the Rossman fold) (Figs. 1, S2–S4, Tables S1, and S2).

A previous model (21) of SIRT6 bound to H3K9MTU was used for initial model building and adjusted locally. The DNA around the histone core was visible from superhelical location (SHL) 0 to SHL 7 on the side SIRT6 was not bound and from SHL 0 to SHL 6.5 on the side SIRT6 was bound. The remaining DNA was not resolved. Core residues of SIRT6 from 2 to 290 were visible. SIRT6 engaged with the nucleosome face, spanning from the acidic patch, across the H2A tail docking domain, to DNA at SHL 6.5 (Figs. 1, S2–S4, Tables S1, and S2). The SIRT6 zinc-binding loop is bound to the acidic patch, whereas the Rossman fold containing the H3K27MTU analog reaches across the nucleosome surface and engages DNA at SHL 6.5.

### Structural comparison of SIRT6 targeting of H3K27 *versus* H3K9 in nucleosomes

**Figure 2:**
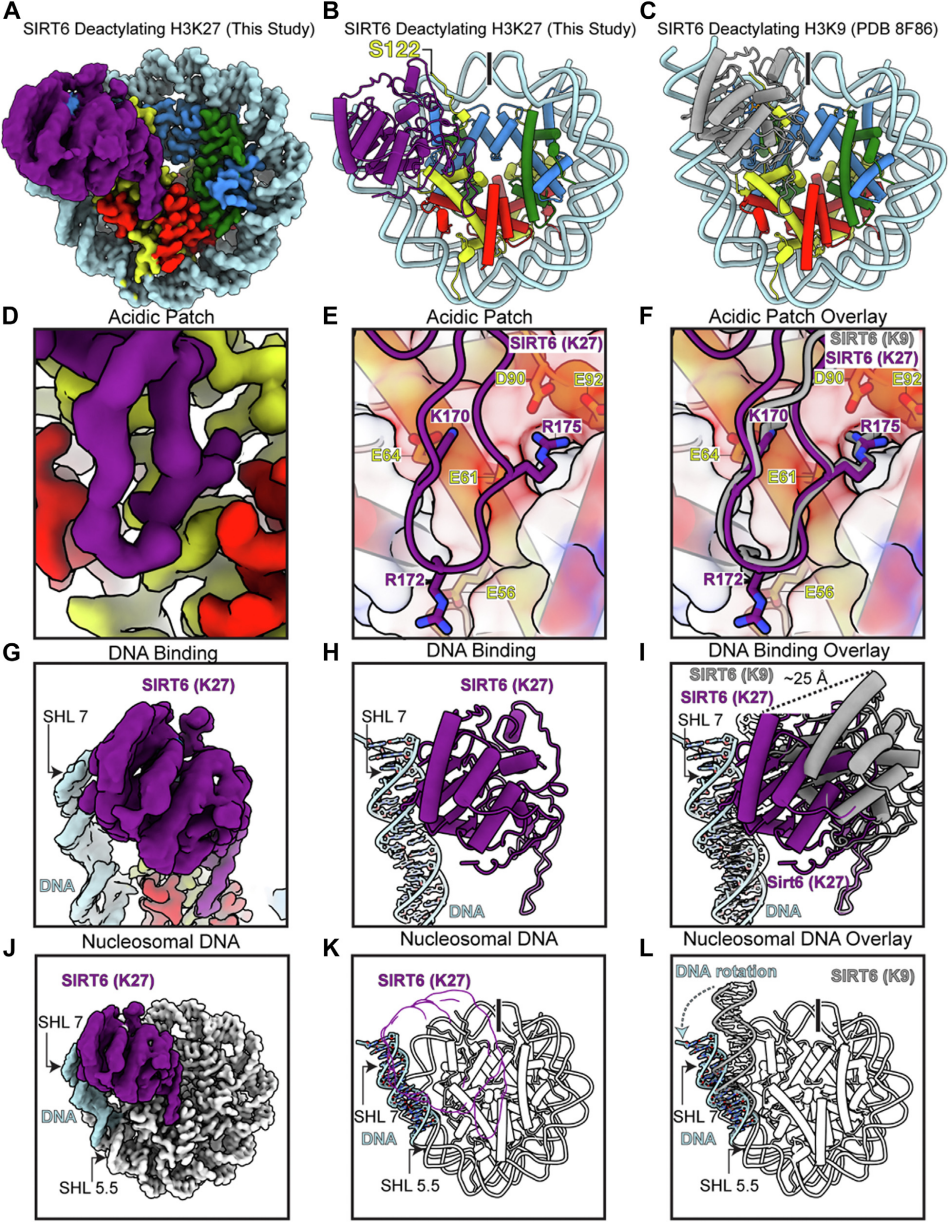
**Comparison between SIRT6 trapped with H3K27MTU and H3K9MTU nucleosomes**. (*A*) Map and (*B*) model of SIRT6 trapped with H3K27MTU nucleosome (this study). *C*, model of SIRT6 trapped with H3K9MTU nucleosome (Protein Data Bank code: 8F86). *D*, Map and (*E*) model close-up interactions of SIRT6-trapped with H3K27MTU nucleosome highlighting acidic patch interactions. *F*, same view as *E* with SIRT6 trapped with H3K9MTU nucleosome, overlay in *gray*. *G*, Map and (*H*) model close-up of the DNA interactions of SIRT6 trapped with H3K27MTU. *I*, same view as *H* with SIRT6 trapped with H3K9MTU nucleosome, overlay in *gray*. *J*, Map and (*K*) model trajectory of the DNA between SHL 5 and SHL 7 in the model of SIRT6 trapped with H3K27MTU nucleosome. *L*, same view as *K* with SIRT6 trapped with H3K9MTU nucleosome, overlay in *gray*. H3K9, histone H3 at lysine 9; H3K27, histone H3 at lysine 27; MTU, methyl-thiourea; SIRT6, sirtuin 6.

Our structural analysis of SIRT6 bound to H3K27- *versus* H3K9-modified nucleosomes reveals similarities and remarkable plasticity in how this enzyme recognizes different nucleosomal substrates (Fig. 2, *A* and *B*). One constant feature across both structures is SIRT6's interaction with the nucleosome acidic patch, where residues K170, R172, and R175 engage with acidic residues on H2A (residues E56, E61, E64, D90, and E92) (Fig. 2, *D*–*F*). The minimal rearrangement in these residues suggests that the acidic patch serves as a conserved anchor point for nucleosome recognition. In contrast to the conserved acidic patch interaction, we observed three major adaptations that enable SIRT6 to access the distinctly positioned H3K27 substrate.

First, the catalytic region of SIRT6, comprised of the two Rossmann folds (residues 25–133 and 195–268), underwent a ∼25 Å movement compared with the H3K9MTU-bound structure (Fig. 2, *G*–*I*). This substantial conformational change demonstrates SIRT6's inherent flexibility in accommodating different histone modification substrates. Second, we observed a shift in SIRT6's DNA engagement. In the H3K9MTU structure, SIRT6 interacts with DNA at SHL 7 (Fig. 2*I*). While these same residues maintain DNA backbone contacts in our H3K27-bound structure, their interaction point shifts toward SHL 6 (Fig. 2, *G* and *H*), indicating precise repositioning of the enzyme's DNA-binding interface in the presence of a substrate that is located closer to the H3 core. Third, we observe increased DNA unwrapping in the H3K27-bound structure to accommodate deacetylation at H3K27. Although both H3K9MTU and H3K27MTU structures show DNA peeling from SHL 5.5 to 7, the H3K27MTU structure exhibits an additional rotation away from the histone core (Figs. 2, *J*–*L* and S4). This increased unwrapping suggests that SIRT6 requires more extensive disruption of histone–DNA contacts to access acetylation marks positioned closer to the histone core. This observation aligns with recent molecular dynamics studies showing that SIRT6-mediated DNA unwrapping enables alternative H3 tail conformations, making histone core–proximal lysine residues (like H3K27) more accessible to the enzyme's active site (26).

Notably, the H2A tail remains unresolved past residue S122 (Fig. 2*B*), suggesting that the H2A tail interaction is not fundamentally required for SIRT6 activity (22). In addition, the absence of visible H3 tail residues near SHL 6.5 indicates distinct H3 tail dynamics between the H3K9 and H3K27 binding modes.

In summary, our structural analysis reveals how SIRT6 exploits inherent flexibility in nucleosome engagement to adapt its binding mode while maintaining key nucleosome interactions. This structural versatility explains how SIRT6 effectively targets multiple histone H3 residues despite their distinct spatial positioning within the nucleosome.

### SIRT6 as an efficient deacylase for H3K9 lactyl and **β**-hydroxybutyryl modifications

**Figure 3:**
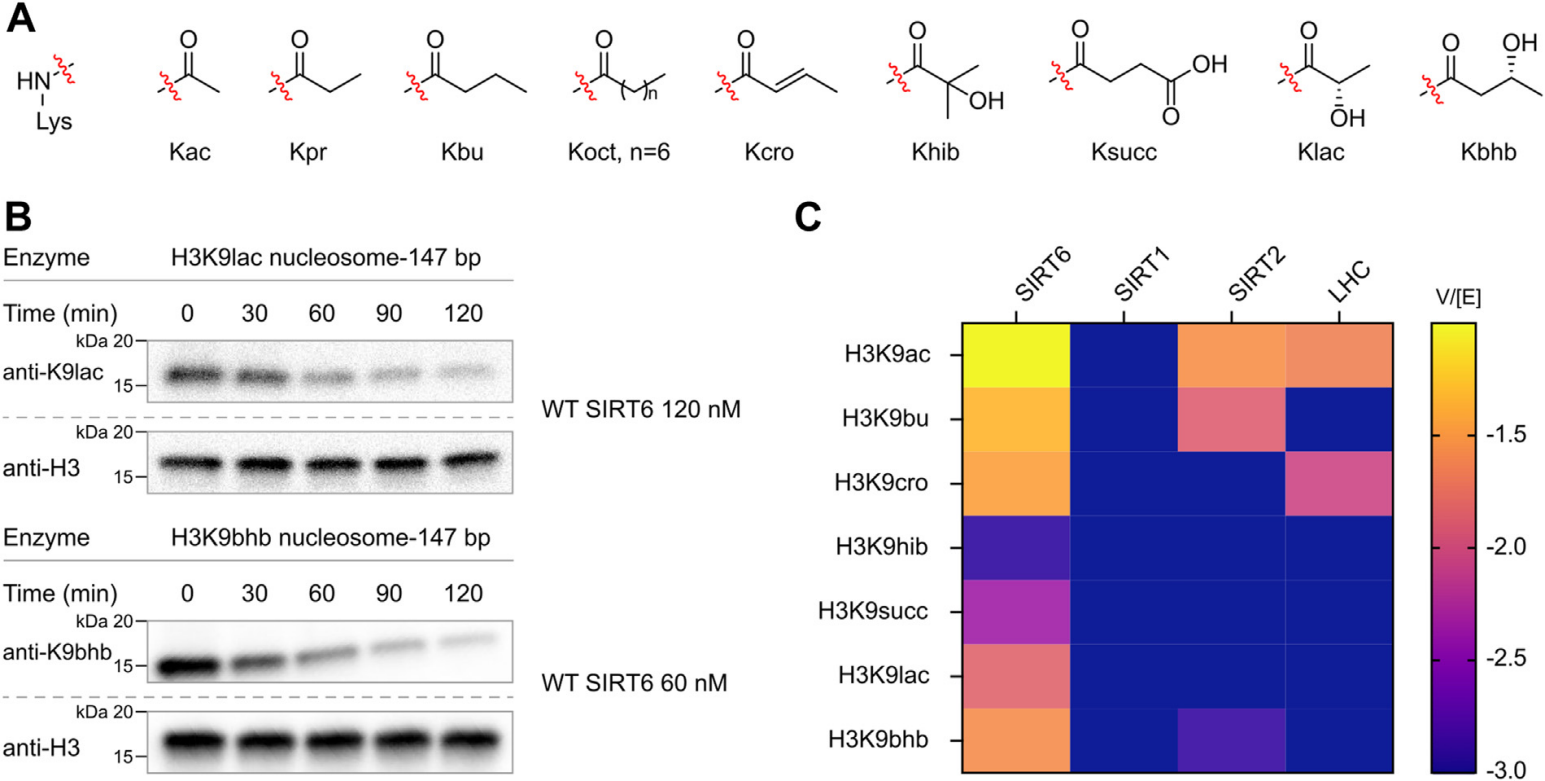
**Deacylation of various H3K9-acyl modifications in nucleosomes by SIRT1, SIRT2, SIRT6, and the LSD1–HDAC1–CoREST (LHC) complex.***A*, structures of the acyl-Lys modifications investigated here. *B*, representative Western blot results for SIRT6 assay with H3K9lac (n = 4) and H3K9bhb (n = 6) nucleosomes. *C*, heatmap for V/[E] (min^−1^) of SIRTs and LHC. H3K9, histone H3 at lysine 9; SIRT, sirtuin.

Recent proteomic studies have uncovered a variety of lysine acylations, with their accumulation closely linked to the metabolic states of both the host and microbiota (27). To further explore the regulation of these modifications, we surveyed enzymatic activity toward various acylated nucleosomal substrates. We elected to focus on the H3K9 position for these experiments because this histone site has previously been found to be the most efficient H3 site for deacetylation (20, 21) and is well established to be targeted by SIRT6 in cellular studies. We exploited a recently developed pipeline that allows for the site-specific installation of these marks using peptide synthesis and sortase-dependent ligation (23). In this approach, the H3-tailless histone octamer is obtained by coexpression and then wrapped with 147 bp DNA to furnish H3-tailless nucleosomes. The engineered sortase cW11 is then used to ligate the requisite H3 tails to the tailless nucleosomes to afford the desired H3-modified nucleosomes. Using this technology, we incorporated propionylation (Kpr), butyrylation (Kbu), octanoylation (Koct), crotonylation (Kcro), lactylation (Klac), α-hydroxyisobutyrylation (Khib), β-hydroxybutyrylation (Kbhb), and succinylation (Ksucc) marks on H3K9 using a nucleosome with 147 bp DNA (Fig. 3*A*). Full-length recombinant WT SIRT6 was tested against the various acylated nucleosome substrates. We utilized a time-dependent Western blot–based deacylation assay and compared the rates of sirtuin family members and histone deacetylase (HDAC) complexes. Enzyme concentrations were optimized to obtain quantitatively reliable deacylation rates under our assay conditions for a given substrate. Prior work demonstrated that the reaction velocities *versus* Sirt6 concentrations are linear in the range employed (21).

In general, the activity of SIRT6 outstripped SIRT1, SIRT2, and the CoREST complex (LSD1–HDAC1–CoREST; LHC) with nearly all acylations. SIRT6 shows clear preferences based on acyl chain length and branching. SIRT6 removes small, nonpolar, nonbranched acylations, such as Kac, Kbu, and Kcro more rapidly than polar and branched acylations like Khib, Ksucc, Klac, and Kbhb (Figs. 3, *B* and *C*, S5, S6, and Table S3). The enzyme removes Kbhb (V/[E] = 0.026 ± 0.0018 min^−1^) ∼3-fold slower than Kac but still at a significantly faster rate than other four-carbon acylations (C4). Notably, the structural isomer Khib is processed 20-fold more slowly (Khib V/[E] = 0.0013 ± 0.00049 min^−1^), highlighting a preference against highly branched substrates. By contrast, Klac (28), which has one less branching methyl substituent than Khib, is processed 10-fold faster (V/[E] = 0.014 ± 0.00085 min^-1^).

The superior activity of SIRT6 toward these polar modifications may link it to multiple metabolic states. For example, elevated lactic acid concentration, a key metabolic product of anaerobic respiration, leads to greater Klac (29). In addition, Kbhb levels correlate with increased β-hydroxybutyrate concentrations, which rise during metabolic ketoacidosis and ketone body metabolism during a ketogenic diet (30).

### G60A SIRT6 mutation affecting deacylation activity

SIRT6 Gly60 falls within the α2–α3 linker of the sirtuin family Rossman fold, which is highly conserved among eukaryotes with ∼90% conservation of the sequence G-I-P-D-F/Y-R (SIRT6 aa 60–aa 65) (31). The α2–α3 linker in related sirtuins can shift 3 to 7 Å, adopting distinct conformations when binding to peptide and NAD cosubstrates, peptide, nicotinamide (NAM), or 2-acyl ADP ribose products (Fig. S7) (22, 32, 33). The flexibility of G60 is believed to contribute to these conformational changes and thus to catalysis.

It was previously observed that a G60A mutation significantly altered cellular metabolism and longevity (25). Peptide activity assays indicated that G60A renders the enzyme catalytically active toward long-chain acylations alone. We previously observed that SIRT6 is much more active toward acetylated nucleosomes than acetylated protein, suggesting that engaging the nucleosome activates SIRT6 for catalysis (21), and could overcome the destabilizing effect of the G60A mutation.

**Figure 4:**
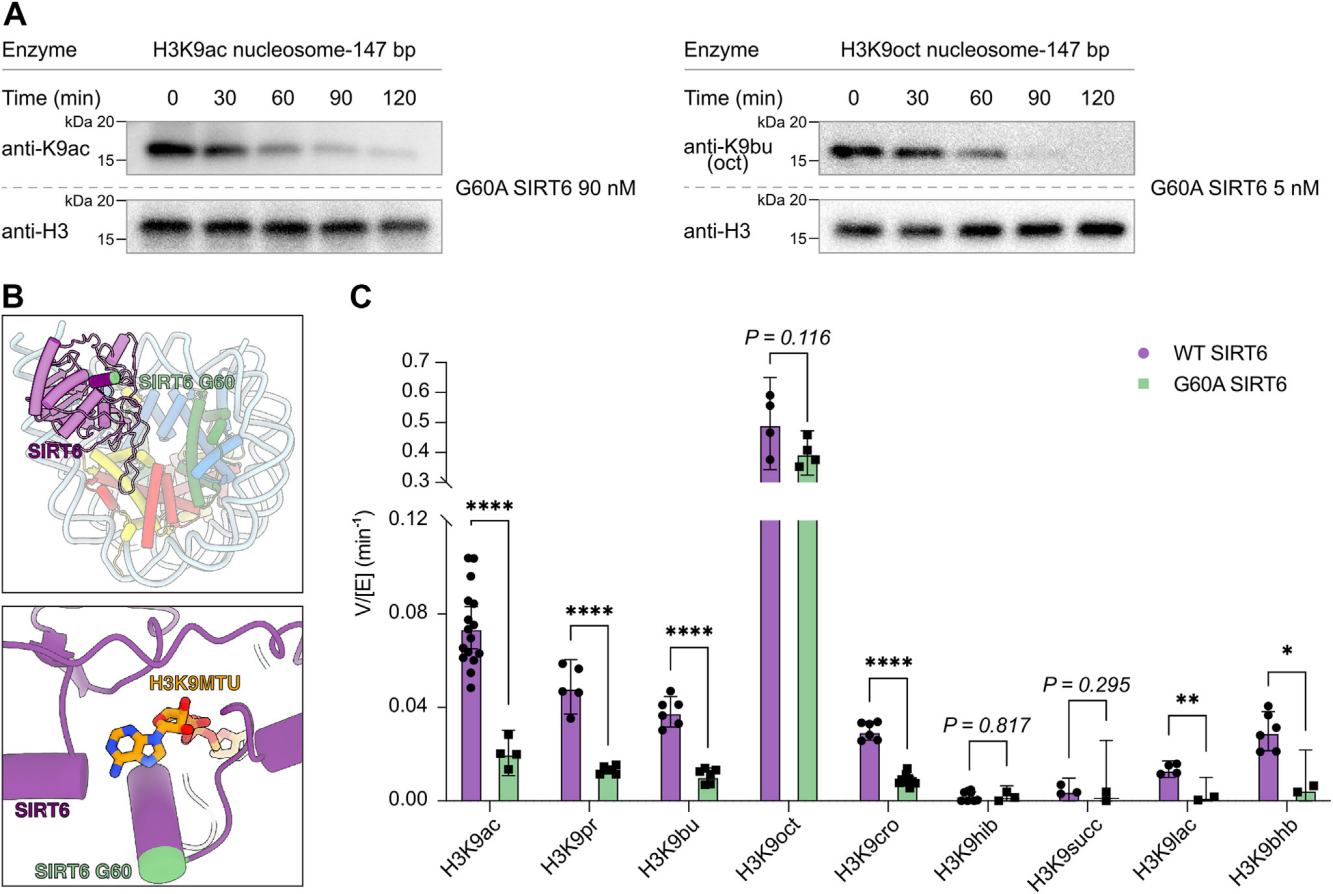
**Deacylation activity of G60A SIRT6 with various H3K9-acyl modifications in nucleosomes.***A*, representative Western blot results for SIRT6 assay on H3K9ac (n = 4) and H3K9oct (n = 6) nucleosomes. *B*, the position of G60 in SIRT6 bound to H3K9MTU (Protein Data Bank code: 8F86) (*top*) and zoomed in structure (*bottom*). *C*, bar graph for V/[E] (mean ± SD min^−1^) of deacylation activity comparing WT SIRT6 and G60A SIRT6 (ns *p* > 0.1; ∗*p* = 0.0061; ∗∗*p* = 0.0019; and ∗∗∗∗*p* < 0.0001; multiple unpaired *t* tests with Holm–Šídák correction; n = 2–10). H3K9ac, histone H3 at lysine 9; MTU, methyl-thiourea; ns, not significant; SIRT6, sirtuin 6.

G60A SIRT6 was cloned, expressed in *Escherichia coli*, and purified following the same protocol as for WT SIRT6 (Fig. S8). Nine different acylated nucleosomes at the H3K9 site, including Kac, Kpr, Kbu, Koct, Kcro, Khib, Klac, Kbhb, and Ksucc, were investigated as substrates. Consistent with peptide substrate assays, G60A demonstrated a preference for long-chain modified nucleosomes over nucleosomes with short-chain acyl modifications. The data indicated that G60A SIRT6 has an approximately fourfold slower rate of deacetylation (V/[E] = 0.020 ± 0.00094 min^−1^) compared with WT (V/[E] = 0.075 ± 0.0044 min^−1^), whereas the deoctanoylation rate (V/[E] = 0.40 ± 0.044 min^−1^) remains similar to that of the WT (V/[E] = 0.46 ± 0.063 min^−1^) (Figs. 4 and S9).

Previous studies assessed the effect of G60A on the apparent affinity for NAD and found that binding was 21-fold weaker in the presence of acetylated peptide cosubstrate, but only ∼1.5-fold weaker in the presence of a long-chain acylated version of the same peptide substrate (25). We found that G60A SIRT6 exhibited a fivefold increase in *K*_*m*_ values for both nucleosome (*K*_*m*_ = 88 ± 27 nM) and NAD substrates (*K*_*m*_ = 757 ± 156 nM), as compared with those previously measured for WT SIRT6 (nucleosome *K*_*m*_ = 17 ± 4.1 nM; NAD *K*_*m*_ = 140 ± 19 nM) (21) (Figs. S10 and S11). These results further support the observation that G60A decreases the apparent affinity for both the acylated substrate and NAD, which reduces its catalytic efficiency (Table S3).

### Sensitivity to proximal histone PTMs

Proteomics data reveal that the histone H3 tail is frequently modified with multiple PTMs (34), leading to instances of PTM crosstalk that affect the action of histone-modifying enzymes (35). Therefore, we investigated whether SIRT6’s deacylation activity could be influenced by local PTM combinations.

Previous enzymology studies have indicated that the presence of a positively charged polar residue (Arg) adjacent to an acetyl-Lys site can stimulate deacetylation, a trend observed in several HDAC1 complexes and sirtuins (36). In this prior work, switching the R8 site residue in H3K9ac nucleosomes to G8, or the G13 residue in H3K14ac nucleosomes to Arg13, resulted in inversion of HDAC1 complex enzymatic selectivity, indicating that the −1 Arg stimulates deacetylation of the neighboring acetyl-Lys (37). The biomedical significance of this sequence is interesting as it is similar to a pattern of germline mutations (R8G/S/C, G14R, and R17G) associated with neurodegeneration (38).

**Figure 5:**
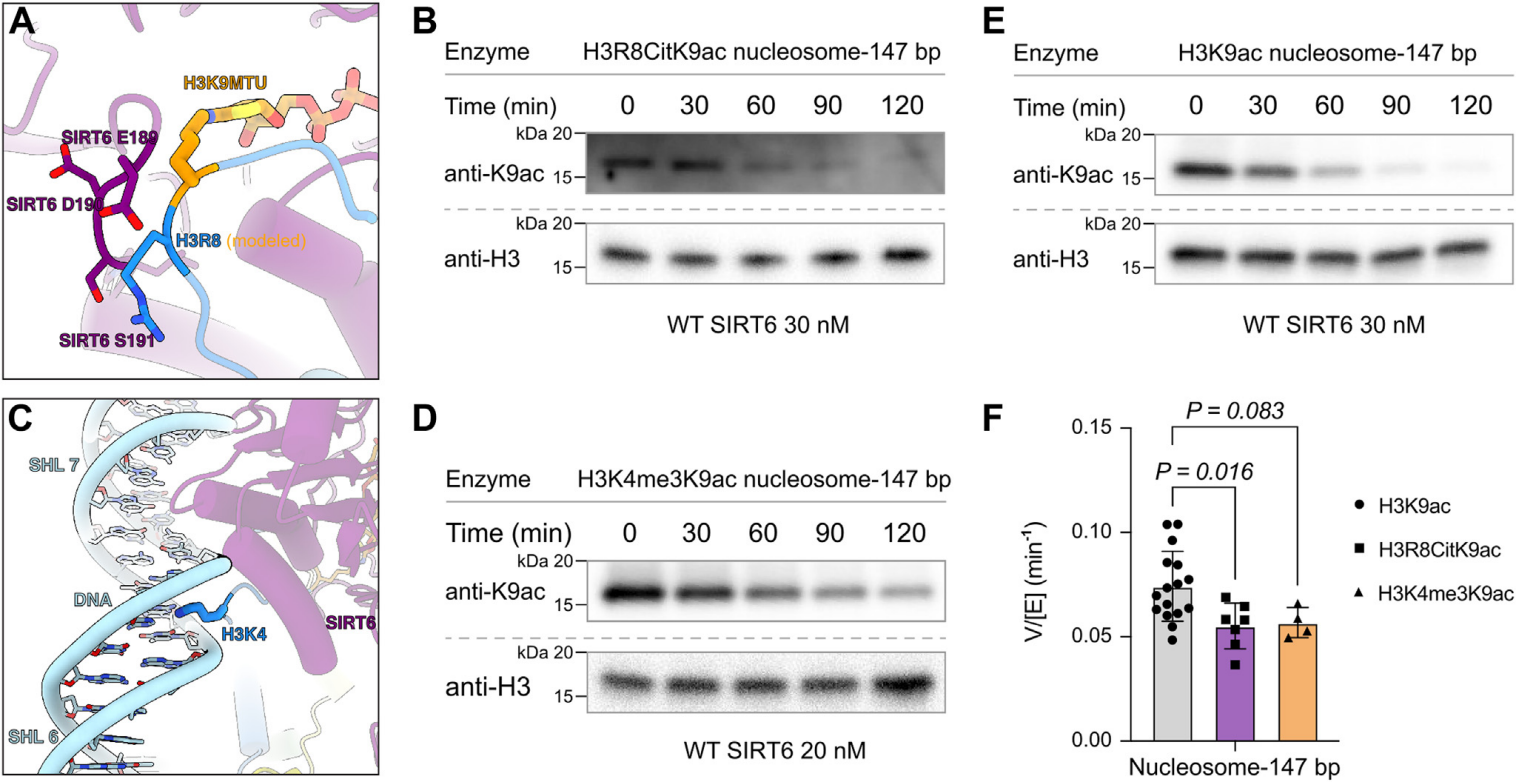
**SIRT6 deacetylation activity with H3K4me3/H3K9ac and H3Cit8/H3K9ac nucleosome substrates.***A*, the position of H3R8 in the structure of SIRT6 trapped with H3K9MTU nucleosome. *B*, representative Western blot results for SIRT6 assay with H3R8Cit/H3K9ac nucleosome substrate (n = 7). *C*, the position of H3K4 in the structure of SIRT6 trapped with H3K9MTU nucleosome. *D*, representative Western blot results for SIRT6 assay with H3K4me3/H3K9ac nucleosome substrate (n = 4). *E*, representative Western blot results for SIRT6 assay with H3K9ac nucleosome substrate (n = 4). *F*, bar graph comparison of SIRT6 V/[E] (mean ± SD min^−1^) for assays in *B*, *D*, and *E* (one-way ANOVA, *post hoc* Dunnett’s multiple comparisons test). H3K9ac, histone H3 at lysine 9; MTU, methyl-thiourea; SIRT6, sirtuin 6.

Enzymatic deimination H3R8 to citrulline (Cit) is also reported and is found in neutrophil extracellular traps, characteristic of rheumatoid arthritis (39). As citrullination removes the arginine-positive charge while maintaining its hydrogen bonding potential, we investigated whether this pathological modification could influence deacetylation at H3K9ac (Fig. 5*A*). We prepared H39Kac nucleosome substrates comodified with the H3R8Cit and tested these in deacetylation assays. The results demonstrated an approximately 30% decrease in deacetylation activity (V/[E] = 0.051 ± 0.0057 min^−1^) compared with the natural H3R8 nucleosomes. This finding indicates that modifications at R8 may help enhance stability of H3K9ac in chromatin (Figs. 5 and S12).

Based on the prior structural analysis of SIRT6 trapped with an H3K9MTU nucleosome, H3K4 is positioned to interact with DNA at its minor groove through charge–charge interactions (Fig. 5*C*). Based on this observation, we hypothesized that H3K4me3 might affect SIRT6’s deacetylation activity on H3K9ac nucleosomes. To examine this, we prepared nucleosome substrates containing both H3K4me3 and H3K9ac on the same histone tail. SIRT6 assays with this nucleosome substrate showed a slight (∼20%), but not statistically significant, decline in deacetylation efficiency for the H3K4me3K9ac nucleosome (V/[E] = 0.062 ± 0.0023 min^−1^) compared with the H3K9ac nucleosome (V/[E] = 0.075 ± 0.0044 min^−1^). These results support prior mutagenesis studies indicating that the apparent H3K4 interaction may not have much influence on SIRT6 catalysis (22) (Figs. 5, S12, and Table S4).

### Small-molecule modulators

**Figure 6:**
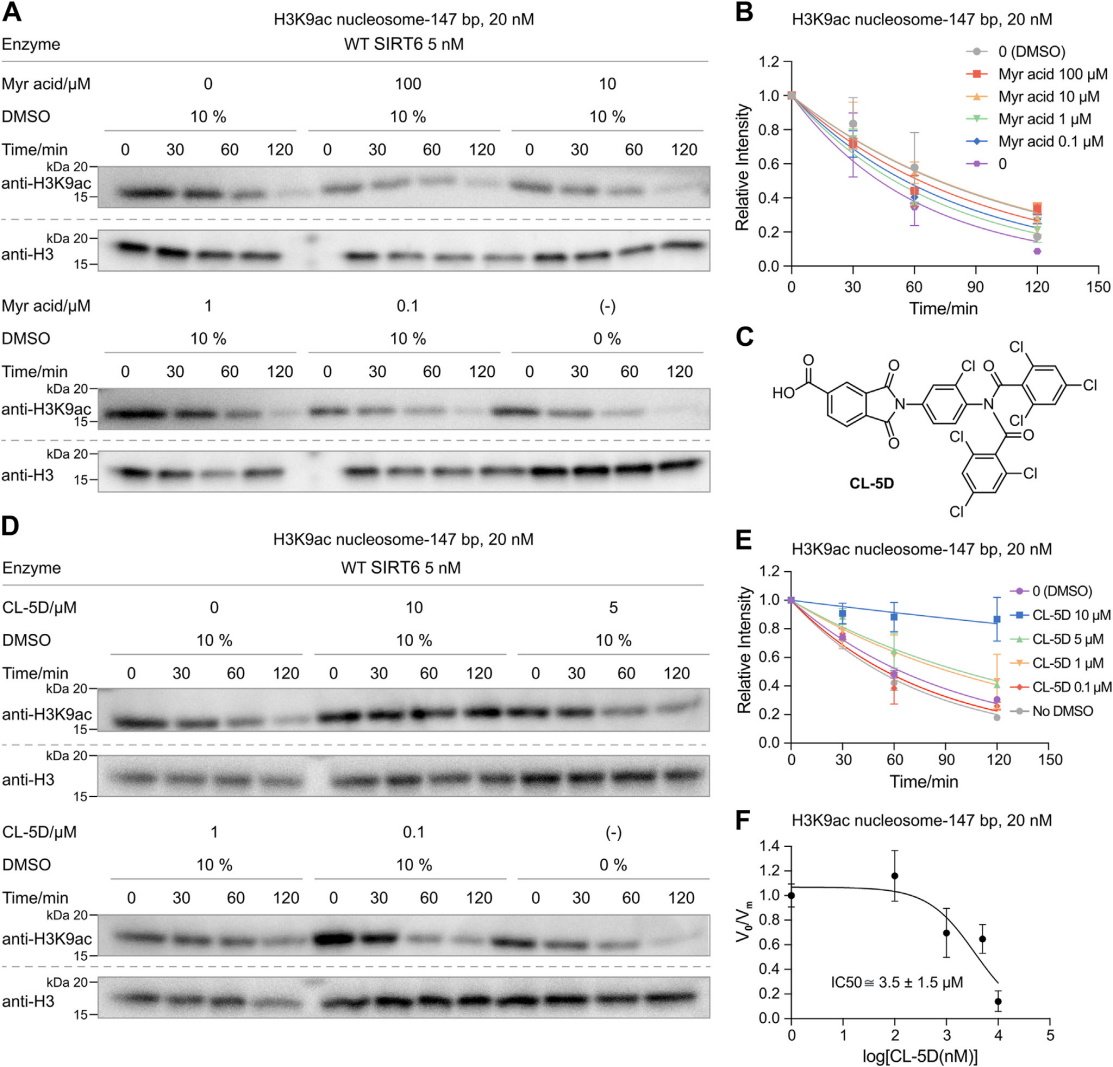
**Effects of myristic (Myr) acid and CL-5D on SIRT6 H3K9ac nucleosome deacetylation.***A*, Western blot analysis of Myr acid effects (n = 4; ∗ indicates ladder lane). *B*, SIRT6 deacetylation rates with different concentrations of Myr acid. *C*, CL-5D structure. *D*, Western blot analysis of CL-5D effects (n = 4; ∗ indicates ladder lane). *E*, SIRT6 deacetylation rates with different concentrations of CL-5D. *F*, IC_50_ determination with CL-5D. Kinetic values shown are ±SD. H3K9ac, histone H3 at lysine 9; SIRT6, sirtuin 6.

Stimulation of SIRT6 activity by small molecules has garnered interest as a potential strategy for extending healthspan (40). Myristic acid, CL-5D, NAM, and MDL-800 have been reported as modulators of SIRT6 activity toward peptides (41). We previously corroborated allosteric activation of SIRT6 with nucleosome substrate by the small molecule MDL-800 (21). Extending the analysis of small molecules in the current study, we found that myristic acid (up to 100 μM) had no effect on SIRT6 deacetylation of H3K9ac nucleosomes, in contrast to prior work on peptide substrates (42) (Figs. 6, *A* and *B* and S13–S15). CL-5D (Fig. 6*C*), an activator with peptide substrates (43), unexpectedly inhibited H3K9ac nucleosome deacetylation, with an IC_50_ of ∼3.5 μM in our hands (Figs. 6, *D*–*F*, S16, and S17). We conducted peptide-based assays and confirmed that CL-5D stimulates activity with peptide substrates (Fig. S18, *C* and *D*). Interestingly, when using full-length semisynthetic histone protein H3K9ac as substrate, CL-5D showed no significant effect on deacetylation activity, either as an activator or as an inhibitor (Fig. S18, *A* and *B*). These findings reveal the critical role of substrate context in interpreting pharmacological impacts on SIRT6 activation.

The well-known generic inhibitor, NAM, was confirmed to inhibit SIRT6 nucleosome deacetylation, with an IC_50_ of ∼8.5 mM, in the same range as previously reported (44). Notably, we found that CL-5D exhibits a much stronger inhibitory effect on SIRT6 with nucleosome substrates than the generic inhibitor NAM (Fig. S19). To further analyze the potential inhibition mechanism of CL-5D, we performed an additional assay to test the competition between CL-5D and MDL-800. MDL-800 previously was determined to have an EC_50_ of ∼3.5 μM as a deacetylation activator on H3K9ac nucleosome substrates in our hands. In the presence of MDL-800 (100 μM), we found that CL-5D exhibited a higher IC_50_ of ∼9.0 μM (Fig. S20) compared with ∼3.5 μM in the absence of MDL-800. This suggests that MDL-800 and CL-5D may compete for an overlapping allosteric-binding site on SIRT6 (Table S5).

## Discussion

This study reveals how SIRT6 accommodates nucleosomal substrates with a diversity of H3 acylations by specifically engaging the nucleosome while maintaining active site plasticity. These results begin to illuminate how SIRT6 can play versatile roles across a variety of biological processes (45, 46). We and others recently found that SIRT6 is an efficient eraser of acylations in nucleosomes spanning H3K9 to H3K27 (21, 26), although this appears sensitive to ionic strength (22). Using cryo-EM, we captured SIRT6 poised to deacetylate a nucleosome at H3K27ac, revealing that different adaptations are required for deacetylation of H3K9 and H3K27. Notably, when engaging H3K27, SIRT6 binds the nucleosome surface from the acidic patch toward SHL 6.5, and in doing so, facilitates the unwrapping of DNA from the histone core. This binding mode is unusual among factors recognizing H3 modifications near the nucleosome DNA. PRC2, for example, also recognizes and acts on H3K27 sites, yet employs a more subtle binding mode that does not require nucleosomal DNA unwrapping for its activity (47). The deacylase SIRT7 acts on H3K36 site while pinning DNA to the octamer, rather than unwrapping it (48). Structural comparison of the H3K9ac- and H3K27ac-trapped complexes with SIRT6 showed conserved nucleosome acidic patch engagement. However, SIRT6 shows divergent conformations to accommodate these different histone marks, demonstrating that SIRT6 possesses extensive plasticity to target distinct H3 lysine sites and pointing to a broad versatility of SIRT enzymes to successfully erase a broad range of acylation marks.

Extending enzymatic analysis of SIRT6, we examined a range of substrates acylated on the favored H3K9 position and observed a preference for nonpolar, nonbranched acylations over polar and branched acylations. Prior crystallographic analyses of SIRT6 with long-chain acylated substrates revealed a hydrophobic channel flanking the conserved NAD-binding α2–α3 linker. Within that loop, I61, P62, and F64 make up part of the hydrophobic channel and position D63, F64, and R65 to bind NAD. These residues lose structure in the absence of bound acylation, NAD or ADPr, which is accompanied by rotation of the G60 backbone (22, 26). The selectivity for long-chain acylations imparted by G60A, along with the ∼5-fold reduction in apparent affinity for both acetylated substrates and NAD, suggests that the flexibility of G60 is critical for repositioning the α2–α3 linker for substrate binding, catalysis, and product release.

Flexibility within the α2–α3 linker appears coupled to flexibility of the entire N-terminal portion of the SIRT6 active site (M1–A13; G60–G69; and G77–T84). This may also contribute to tolerance for branched and polar acylations. We find that SIRT6 removes branched acylations (Fig. 3*A*) substantially faster than other sirtuins and one of the faster HDAC1 complexes (Fig. 3*C*). The latter were previously shown to remove Kbhb *in vitro* (SIRT1 and SIRT2) and *in vivo* (HDAC1/2) (30), suggesting a role for SIRT6 in the endogenous removal of these modifications.

Evaluating the local peptide sequence has been observed to influence HDAC activity (21, 37), leading us to evaluate how proximal combinatorial histone modifications could alter SIRT6 activity. We observed that citrullination of Arg8 moderately shelters H3K9ac against deacetylation by SIRT6. Charge neutralization by citrullination contributes to chromatin decondensation during formation of neutrophil extracellular traps, and crosstalk between charge neutralizing modifications could contribute to this process.

SIRT6 exhibits sevenfold faster removal of the eight-carbon fatty acyl octanoyl group over acetyl with nucleosome substrates, which is nonetheless much smaller than was observed for peptides (42). This suggests that the stimulatory effect of nucleosome recognition ameliorates the critical requirement for fatty acids in more weakly binding peptides. In future work, it will be interesting to extend these studies to additional H3 sites including H3K27.

The differences in activity observed here toward nucleosome and peptide substrates have important implications for pharmacological development, as illustrated by the divergent activities of small-molecule modulators on SIRT6’s deacylase activity across these two substrate classes. Notably, the majority of SIRT6 activators and inhibitors reported to date bind in a similar region of the flexible N terminus (22). We observe that nucleosome binding depends on interactions with both DNA and the acidic patch, which generally enhances SIRT6 deacetylation (21), while mitigating both the inhibitory effects of G60A and the stimulatory effects of myristic acid. This suggests that nucleosome binding supports a set of active site reorganizations required for NAD binding, catalysis, and product release. This may in turn begin to explain the observation that MDL-800 activates SIRT6 on nucleosome substrates, CL-5D shows the inverse effect, acting as an inhibitor, even though both stimulate SIRT6 with peptide substrates. While the structural basis for these differences is not yet clear, our findings highlight the value of analyzing a diverse range of substrates (49) to obtain a holistic understanding of SIRT6’s biological functions (50).

## Experimental procedures

### Protein purification of WT SIRT6 and G60A SIRT6

WT SIRT6 and G60A SIRT were expressed following protocols previously established (21). Following transformation with the requisite DNA plasmids, the LOBSTR *E. coli* strain derived from Rosetta (DE3) was cultured at 37 °C until an absorbance of 0.6 at 600 nm was achieved, upon which overexpression was induced by the addition of 0.5 mM isopropyl β-d-1-thiogalactopyranoside. Subsequently, cells were cultured for an additional 18 h at 25 °C. The cells were then lysed, and proteins were purified using nickel–nitrilotriacetic acid resin. The eluted proteins were concentrated utilizing a spin concentrator (Amicon, 10 kDa molecular weight cutoff, 4000 rpm, 4 °C) and subsequently incubated with tobacco etch virus protease overnight at 4 °C to remove the His-tag. The flow-through was applied to a Heparin column (Cytiva, 1 ml), where pure fractions were collected and further concentrated. The material underwent additional purification *via* size-exclusion chromatography using a Superdex200 Increase 10/300 Gl column (Cytiva). The purest fractions obtained were pooled and concentrated. All constructs of SIRT6 were aliquoted, flash-frozen in liquid nitrogen, and stored at −80 °C until further use.

### Peptide synthesis

All peptides were prepared by Fmoc-based solid-phase peptide synthesis using Rink Amide AM resin (EMD Millipore), facilitated by a Prelude automated peptide synthesizer (Gyros Protein Technologies) as described previously (36). For a 0.2 mmol scale resin, 0.8 mmol (4 eq) Fmoc-amino acid in 4 ml dimethyl formamide, 0.75 mmol (3.75 eq) HATU, and 1.6 mmol (8 eq) *N*-methylmorpholine in 4 ml dimethyl formamide were sequentially added to the reaction vessel and subjected to double coupling for 90 min each time. Cleavage of the peptide from the resin and removal of the side-chain protecting groups were achieved by the addition of reagent B (5% water, 5% phenol, 2.5% triisopropylsilane, 87.5% trifluoroacetic acid) followed by 90 to 180 min of agitation. Crude peptides were purified *via* reverse-phase HPLC using a C18 semipreparative column (Varian Dynamax Microsorb 100, 250 × 21.4 mm). Purified fractions (>95% pure) were identified by MALDI-TOF MS (Dana Farber Cancer Institute Molecular Biology Core Facilities, 4800 MALDI TOF/TOF; Applied Biosystems/MDS Sciex) or electrospray ionization–mass spectrometry (Q Exactive, Thermo Scientific). The identified purified fractions were then combined, lyophilized, and stored as dry powders at −80 °C. The synthesized peptides were characterized by electrospray ionization–mass spectrometry (Q Exactive, Thermo Scientific) and deconvoluted using UniDec.

### Reconstitution of modified nucleosomes

Nucleosome reconstitution and installation of H3 PTMs was performed as reported previously (37). Briefly, purified H3 tailless histone octamer was combined with Widom 601 DNA (147 bp core sequence or 185 bp sequence with 20 bp flanking on either side) in high salt reconstitution buffer (2 M KCl, 10 mM Tris, pH 7.9 at 25 °C, 1 mM EDTA, and 10 mM DTT). The optimal molar ratio was determined by test scale reconstitution (generally 1.1:1–1.3:1 octamer:DNA). The mixture was incubated for 30 min on ice, then transferred to a dialysis bag, and placed in 1 l of high salt reconstitution buffer (2 M KCl, 10 mM Tris, pH 7.9 at 25 °C, 1 mM EDTA, and 1 mM DTT). Linear salt gradient dialysis into low salt reconstitution buffer (10 mM Tris, pH 7.9 at 25 °C, 150 mM KCl, 0.1 mM EDTA, and 1 mM DTT) was carried out over 30 to 48 h using a dual channel peristaltic pump with a constant influx/efflux rate of 2 ml/min, after which the sample was subjected to a final round of dialysis against low salt reconstitution buffer.

### Ligation of histone tails

Histone tail peptides dissolved in Milli-Q water were mixed with H3 tailless nucleosomes and cW11 sortase in reaction buffer (40 mM Tris, pH 7.5 at 25 °C, 150 mM NaCl/KCl, 5 mM CaCl_2_, and 1 mM DTT; listed concentrations are final and should take into consideration the buffer composition of both nucleosome and sortase). Generally, histone tail peptide (49.5 μM) and nucleosome (1.65 μM) were mixed first, and cW11 sortase (200 μM) was added to initiate the reaction. The reaction was moved to a 37 °C incubator for at least 4 h, then quenched by the addition of NaCl to a final concentration of 250 mM, and salmon sperm DNA (1:1 mass:mass to nucleosome DNA). Insoluble material was removed by centrifugation (5 min, 21.1 k × *g*), and the supernatant was purified by weak anion exchange HPLC (TSKgel DEAE-5PW, 7.5 mm ID × 7.5 cm, 10 μM, TOSOH; mobile phase A: 10 mM Tris, pH 7.9 at 25 °C, 150 mM KCl, 0.5 mM EDTA; mobile phase B: 10 mM Tris, pH 7.9 at 25 °C, 600 mM KCl, 0.5 mM EDTA; gradient: 0–22% B in 3 min, 22% B for 7 min, 22–49% B in 1 min, 49–65% B in 21 min, 65–100% B in 1 min, 100% for 10 min, 100–0% B in 1 min). Ligated products (eluting in the 49–65% B portion of the gradient) were collected and diluted to reduce the concentration of KCl (1:1 v:v; 10 mM Tris, pH 7.5 at 25 °C, 1 mM DTT), then concentrated (Amicon, 10 kDa molecular weight cutoff). Concentrated samples were dialyzed once against dilution buffer at 4 °C and then into storage buffer at 4 °C (10 mM Tris, pH 7.5 at 25 °C, 25 mM NaCl, 1 mM DTT, and 20% glycerol). If needed, nucleosomes were further concentrated to >5 μM, then aliquoted and flash frozen, before storing at −80 °C until use in assays. Nucleosome integrity was confirmed by native TBE gel (4–20%), and ligation product identities were confirmed by dilution into denaturing buffer (7 M guanidine HCl, 20 mM Tris [pH 7.5] at 25 °C, final concentration ∼1 μM) followed by LC–MS (Thermo Q Exactive mass spectrometer; Thermo Vanquish LC; mAbPac 2.1 mm ID x 100 mm, 4 μM; mobile phase A: water + 0.1% formic acid; mobile phase B: acetonitrile + 0.1% formic acid; 0% B for 1 min, 25–55% B for 17 min) (Fig. S20).

### Sample preparation for cryo-EM analysis

SIRT6 (625 nM) and H3K27MTU 185 bp nucleosome (250 nM) were incubated at 37 °C for 120 min in binding buffer (50 mM Hepes [pH 7.5], 150 μM NAD^+^, 1 mM DTT, and 100 mM NaCl). A sample (24 μl) was mixed with 3 μl cross-link buffer (50 mM Hepes [pH 7.5], 1 mM Tris(2-carboxyethyl)phosphine hydrochloride, 100 mM NaCl, and 0.9% glutaraldehyde) for 10 min at 4°C, followed by 3 μl cross-link quench buffer (50 mM Hepes [pH 7.5], 1 mM TCEP, 100 mM NaCl, 24 mM aspartate, and 20 mM lysine) for 10 min at 4 °C. Samples were then dialyzed for 3 h in dialysis buffer (50 mM Hepes [pH 7.5], 1 mM TCEP, and 100 mM NaCl). Quantifoil R2/1, 200 mesh copper grids were glow discharged using a Pelco Easiglow plasma discharge system for 30 S at 15 mA. The sample (4 μl) was applied to grids, blotted with a Vitrobot Mark IV (FEI) at 5 °C and 100% humidity with a wait time of 8 s, blot time 5 s, and blot force of 8, and vitrified with liquid ethane (Fig. S22).

### Cryo-EM data collection and image processing

Cryo-EM data were collected on a ThermoFisher Titan Krios operating at 300 keV, equipped with a Gatan K3 direct electron detector and a Gatan Bioquantum GIF energy filter, using SerialEM. Data were acquired at a pixel size of 0.83 Å with a defocus range between −0.9 and −1.9 μm. Movies consisting of 36 frames were collected with an exposure time of 2.28 s at an electron flux of 21.870 e^−^ Å^−2^ s^−1^, resulting in a total exposure of 49.80 e^-^ Å^-2^. Initial image processing was performed using cryoSPARC (Structura Biotechnology Inc; version 3.2.0). Movies were aligned using cryoSPARC Live Patch Motion Correction, followed by contrast transfer function (CTF) estimation. of the 23,279 micrographs collected, micrographs with CTF less than 4.5 Å were excluded. The cryoSPARC Blob Picker tool selected 10,305,397 particles from the remaining 19,863 micrographs. Particles were then extracted at a box size of 360 pixels. The data were divided into three sets, and identical heterogeneous refinement jobs were performed with four *ab initio* models as inputs. Each heterogeneous refinement yielded a clear nucleosome–SIRT6 class. The datasets were subsequently recombined, and another round of heterogeneous refinement was conducted using four *ab initio* classes as inputs, again producing a clear nucleosome–SIRT6 class. This class underwent further classification through heterogeneous refinement with three classes to enrich for SIRT6. The best classes were then subjected to global and local CTF refinement followed by nonuniform refinement. The resulting map exhibited high-resolution features for histones but lacked high-resolution information for SIRT6 but provided unambiguous secondary structure elements. Consequently, this class underwent additional heterogeneous refinement with four classes. One resulting class, comprising 304,734 particles, resulted in a 3.0 Å reconstruction after nonuniform refinement (Fig. S1 and Table S1). A mask encompassing the SIRT6 Rossman folds was created in RELION, followed by two rounds of masked classification without image alignment. The final class, containing 124,274 particles, was refined to 3.2 Å after nonuniform refinement in cryoSPARC (map A). The maps were postprocessed using DeepEMhancer in cryoSPARC (Fig. S2).

### Model building and figure preparation

Initial structures of the SIRT6–nucleosome complex (Protein Data Bank [PDB] code: 8F86) were rigid body docked into the electron density map. Local adjustments of the structure were performed using Coot (MRC-LMB; version 0.9.7). Extranucleosomal DNA on the SIRT6 binding side was modeled in ChimeraX (UCSF; version 1.8) and subsequently refined in Coot (version 0.9.7). DNA on the opposite side of the nucleosome (opposite the SIRT6 binding side) was only partially visible from approximately SHL 4 to SHL 7 and was retained from PDB (code: 8F86). The NAD analog was constructed in PyMol (Schrodinger; version 2.5.4) with restraints obtained from ELBOW in PHENIX (version 1.20). Final models were subjected to real space refinement in PHENIX (version 1.20) using map A, and the restraints were generated from the ELBOW job. All figures, including cryo-EM maps and structural models, were prepared using ChimeraX (version 1.8). Graphs were plotted using GraphPad Prism (version 10.4; GraphPad Software, Inc), and final figures were assembled in Adobe Illustrator (Fig. S3 and Table S2).

### Nucleosome deacylation activity assays

All nucleosome substrates (final concentration of 100 nM) were diluted in the SIRT6 reaction buffer (50 mM Hepes, pH 7.5, 1 mM DTT, 0.2 mg/ml bovine serum albumin, and 1 mM NAD). Reactions were initiated by the addition of WT SIRT6 or G60A SIRT6 (concentrations as indicated in the figures), followed by incubation at 37 °C. Samples were taken at 0, 30, 60, 90, and 120 min. Aliquots were resolved on a 4% to 20% gradient SDS-PAGE gel and analyzed *via* Western blotting using site-specific antibodies for acylated H3 (pan Klac, a kind gift from Dr Yingming Zhao, University of Chicago; H3K9bhb, PTMbio, catalog no.: PTM-1250) and total H3 (Abcam; catalog no.: ab1791). Membranes were washed, treated with ECL substrate reagent (Bio-Rad), and visualized using a G:mini gel imager (Syngene). Band intensity was quantified using ImageJ (imagej.nih.gov/ij/). Relative intensity values were calculated by normalizing against the intensity at time 0, followed by fitting to a single-phase exponential decay curve with constraints Y0 = 1 and Plateau = 0 (GraphPad Prism 9). Each data point represents at least two replicates. The kinetic parameter V/[E] was calculated using GraphPad Prism 9 (Figs. S4, S5, S7, and S11). All *K*_*m*_ measurements were performed using similar procedures (Figs. S9, S10, and Table S3–S5).

### Nucleosome deacylation assays in the presence of CL-5D, myristic acid, and NAM

CL-5D (Aobious; catalog no.: #AOB11034) was initially prepared as a stock solution in dimethyl sulfoxide at a concentration of 10 mM, followed by serial dilution to final concentrations of 10, 5, 1, and 0.1 μM. Deacylation assays with WT SIRT6 were performed as described previously. To initiate the deacetylation reaction, 20 nM of H3K9ac 147 bp nucleosome was added. Samples were taken from the reaction mixtures at various time points (0, 30, 60, and 120 min) and processed and analyzed as described previosuly. The final rates (V/[E] ± SD) for CL-5D = 10, 5, 1, and 0.1 μM were calculated as described previously and normalized against the control (V/[E] ± SD) CL-5D = 0, dimethyl sulfoxide, providing the SD for each measurement (Figs. S15 and S16). The myristic acid and NAM inhibition assays were performed similarly.

### Fluor de Lys inhibition assays

The Fluor de Lys assay was performed according to the manufacturer’s protocol (BPS Lifesciences; catalog no.: 50087). WT SIRT6 was diluted in SIRT6 reaction buffer (50 mM Hepes, pH 7.5, 1 mM DTT, 0.2 mg/ml bovine serum albumin, and 1 mM NAD) to prepare a 45 μM stock solution. The sirtuin peptide substrate was similarly diluted in the same buffer to obtain a stock solution of 500 μM. For the assay, a final concentration of 50 μM substrate was mixed with several concentrations of SIRT6 (0.5, 1.5, and 4.5 μM) in separate wells of a black 96-well plate (MicroWell, Thermo; catalog no.: 267342). CL-5D was added to the corresponding wells to achieve a final concentration gradient of 1000, 100, 10, and 1 μM. A well containing only the reaction buffer served as the negative control. The plate, covered with aluminum foil, was incubated at 37 °C for 30 min. Subsequently, Sirt2 developer (2×) was added to achieve a final concentration of 1×, followed by an additional incubation for 15 min at room temperature. Fluorescence intensities were measured using a microtiter-plate reading fluorimeter (BioTek Cytation 5) with excitation at 365 nm and emission at 450 nm wavelengths. Data were analyzed by plotting linear fits after subtracting the negative control using GraphPad Prism, with at least two replicates for each data point (Fig. S17).

## Data availability

The 3D cryo-EM maps of the Sirt6-bound nucleosome 3.2 Å average resolution have been deposited in the Electron Microscopy Data Bank under accession codes EMD-48086, and the associated atomic model has been deposited in the PDB under accession code 9EIL. All data are available from the authors upon reasonable request.

## Supporting information

This article contains supporting information.

## Conflict of interest

The authors declare that they have no conflicts of interest with the contents of this article.
