# Supplementary material for: Structural and enzymatic plasticity of SIRT6 deacylase activity
Source: J Biol Chem. 2025 Mar 25;301(5):108446. doi: 10.1016/j.jbc.2025.108446 (PMC12051053; doi:10.1016/j.jbc.2025.108446)
Supplement: Supporting information [file mmc1.pdf]

Structural and Enzymatic Plasticity of SIRT6 Deacylase Activity

Zhipeng A. Wang[#1,2], Jonathan Markert[#3], Samual D. Whedon[#1], Maheeshi Yapa Abeywardana[1], Xinlei Sheng[4], Eunju Nam[1], Kwangwoon Lee[1], Maggie Chen[1], Amanda Waterbury[1], Yingming Zhao[4], Lucas Farnung[*,3], Philip A. Cole[*,1]

1. Division of Genetics, Department of Medicine, Brigham and Women's Hospital, Boston, Massachusetts 02115, United States; Department of Biological Chemistry and Molecular Pharmacology, Harvard Medical School, Boston, Massachusetts 02115, United States;

2. Desai Sethi Urology Institute, Sylvester Comprehensive Cancer Center, University of Miami Miller School of Medicine, Miami, FL 33136, United States;

3. Department of Cell Biology, Blavatnik Institute, Harvard Medical School, Boston, Massachusetts 02115, United States;

4. Ben May Department of Cancer Research, The University of Chicago, Chicago, Illinois 60637, USA;

# These authors contribute equally.

Primers

1. G60A SIRT6-Q5-F
5'- CACTGCCTCTGCGATCCCCGACTTCAGG-3'
G60A SIRT6-Q5-R
5'- CTGATGCCGGCACCCGTG-3'

Plasmids and constructs

1. The full-length SIRT6 (aa 1-355) was constructed in a previous study using a pET28a vector (kanamycin resistant), with a TEV cleavage site positioned between the His-tag and the SIRT6 sequence[1]. An N-terminal cysteine residue was retained to allow for potential future chemical modifications.

His6-TEV-Cys-SIRT6 (aa 1-355) sequence (*His tag is in italics*, TEV sequence underlined)

ATGGGCAGCAGCCATCATCATCATCATCACAGCAGCGGCCTGGTGCCGCGCGGCAGCCAT
GAAAACCTGTATTTTCAGTGCTCGGTGAATTACGCGGCGGGGCTGTCGCCGTACGCG
GACAAGGGCAAGTGCGGCCTCCCGGAGATCTTCGACCCCCCGGAGGAGCTGGAGCG
GAAGGTGTGGGAACTGGCGAGGCTGGTCTGGCAGTCTTCCAGTGTGGTGTTCCACAC
GGGTGCCGGCATCAGCACTGCCTCTGGCATCCCCGACTTCAGGGGTCCCCACGGAGT
CTGGACCATGGAGGAGCGAGGTCTGGCCCCCAAGTTCGACACCACCTTTGAGAGCG
CGCGGCCCACGCAGACCCACATGGCGCTGGTGCAGCTGGAGCGCGTGGGCCTCCTC
CGCTTCCTGGTCAGCCAGAACGTGGACGGGCTCCATGTGCGCTCAGGCTTCCCCAGG
GACAAACTGGCAGAGCTCCACGGGAACATGTTTGTGGAAGAATGTGCCAAGTGTAA
GACGCAGTACGTCCGAGACACAGTCGTGGGCACCATGGGCCTGAAGGCCACGGGCC
GGCTCTGCACCGTGGCTAAGGCAAGGGGGCTGCGAGCCTGCAGGGGAGAGCTGAGG
GACACCATCCTAGACTGGGAGGACTCCCTGCCCGACCGGGACCTGGCACTCGCCGAT
GAGGCCAGCAGGAACGCCGACCTGTCCATCACGCTGGGTACATCGCTGCAGATCCGG
CCCAGCGGGAACCTGCCGCTGGCTACCAAGCGCCGGGGAGGCCGCCTGGTCATCGTC
AACCTGCAGCCCACCAAGCACGACCGCCATGCTGACCTCCGCATCCATGGCTACGTT
GACGAGGTCATGACCCGGCTCATGAAGCACCTGGGGCTGGAGATCCCCGCCTGGGAC
GGCCCCCGTGTGCTGGAGAGGGCGCTGCCACCCCTGCCCCGCCCGCCCACCCCCAA
GCTGGAGCCCAAGGAGGAATCTCCCACCCGGATCAACGGCTCTATCCCCGCCGGCCC
CAAGCAGGAGCCCTGCGCCCAGCACAACGGCTCAGAGCCCGCCAGCCCCAAACGGG
AGCGGCCCACCAGCCCTGCCCCCCACAGACCCCCCAAAAGGGTGAAGGCCAAGGCG
GTCCCCAGC

His6-TEV-Cys-SIRT6 (aa 1-355) amino acid sequence (*His tag is in italics*, TEV sequence underlined)

*MGSSHHHHHHSSGLVPRGSH*ENLYFQCSVNYAAGLSPYADKGKCGLPEIFDPPEELERKV
WELARLVWQSSSVVFHTGAGISTASGIPDFRGPHGVWTMEERGLAPKFDTTFESARPTQ
THMALVQLERVGLLRFLVSQNVDGLHVRSGFPRDKLAELHGNMFVEECAKCKTQYVR
DTVVGTMGLKATGRLCTVAKARGLRACRGELRDTILDWEDSLPDRDLALADEASRNAD
LSITLGTSLQIRPSGNLPLATKRRGGRLVIVNLQPTKHDRHADLRIHGYVDEVMTRLMKH

LGLEIPAWDGPRVLERALPPLPRPPTPKLEPKEESPTRINGSIPAGPKQEPCAQHNGSEPAS
PKRERPTSPAPHRPPKRVKAKAVPS

2. The full-length SIRT6 (aa 1-355) was generated through Q5 mutagenesis using the primers G60A SIRT6-Q5-F and G60A SIRT6-Q5-R.

His6-TEV-Cys-G60A SIRT6 (aa 1-355) sequence (*His tag is in italics*, TEV sequence underlined and **G60A in bold**)

ATGGGCAGCAGCCATCATCATCATCATCACAGCAGCGGCCTGGTGCCGCGCGGCAGCCAT
GAAAACCTGTATTTTCAGTGCTCGGTGAATTACGCGGCGGGGCTGTCGCCGTACGCG
GACAAGGGCAAGTGCGGCCTCCCGGAGATCTTCGACCCCCCGGAGGAGCTGGAGCG
GAAGGTGTGGGAACTGGCGAGGCTGGTCTGGCAGTCTTCCAGTGTGGTGTTCCACAC
GGGTGCCGGCATCAGCACTGCCTCT**GCG**ATCCCCGACTTCAGGGGTCCCCACGGAGT
CTGGACCATGGAGGAGCGAGGTCTGGCCCCCAAGTTCGACACCACCTTTGAGAGCG
CGCGGCCCACGCAGACCCACATGGCGCTGGTGCAGCTGGAGCGCGTGGGCCTCCTC
CGCTTCCTGGTCAGCCAGAACGTGGACGGGCTCCATGTGCGCTCAGGCTTCCCCAGG
GACAAACTGGCAGAGCTCCACGGGAACATGTTTGTGGAAGAATGTGCCAAGTGTAA
GACGCAGTACGTCCGAGACACAGTCGTGGGCACCATGGGCCTGAAGGCCACGGGCC
GGCTCTGCACCGTGGCTAAGGCAAGGGGGCTGCGAGCCTGCAGGGGAGAGCTGAGG
GACACCATCCTAGACTGGGAGGACTCCCTGCCCGACCGGGACCTGGCACTCGCCGAT
GAGGCCAGCAGGAACGCCGACCTGTCCATCACGCTGGGTACATCGCTGCAGATCCGG
CCCAGCGGGAACCTGCCGCTGGCTACCAAGCGCCGGGGAGGCCGCCTGGTCATCGTC
AACCTGCAGCCCACCAAGCACGACCGCCATGCTGACCTCCGCATCCATGGCTACGTT
GACGAGGTCATGACCCGGCTCATGAAGCACCTGGGGCTGGAGATCCCCGCCTGGGAC
GGCCCCCGTGTGCTGGAGAGGGCGCTGCCACCCCTGCCCCGCCCGCCCACCCCCAA
GCTGGAGCCCAAGGAGGAATCTCCCACCCGGATCAACGGCTCTATCCCCGCCGGCCC
CAAGCAGGAGCCCTGCGCCCAGCACAACGGCTCAGAGCCCGCCAGCCCCAAACGGG
AGCGGCCCACCAGCCCTGCCCCCCACAGACCCCCCAAAAGGGTGAAGGCCAAGGCG
GTCCCCAGC

His6-TEV-Cys-G60A SIRT6 (aa 1-355) amino acid sequence (*His tag is in italics*, TEV sequence underlined and **G60A in bold**)

*MGSSHHHHHHSSGLVPRGSH*ENLYFQCSVNYAAGLSPYADKGKCGLPEIFDPPEELERKV
WELARLVWQSSSVVFHTGAGISTAS**A**IPDFRGPHGVWTMEERGLAPKFDTTFESARPTQ
THMALVQLERVGLLRFLVSQNVDGLHVRSGFPRDKLAELHGNMFVEECAKCKTQYVR
DTVVGTMGLKATGRLCTVAKARGLRACRGELRDTILDWEDSLPDRDLALADEASRNAD
LSITLGTSLQIRPSGNLPLATKRRGGRLVIVNLQPTKHDRHADLRIHGYVDEVMTRLMKH
LGLEIPAWDGPRVLERALPPLPRPPTPKLEPKEESPTRINGSIPAGPKQEPCAQHNGSEPAS
PKRERPTSPAPHRPPKRVKAKAVPS

Figure SI

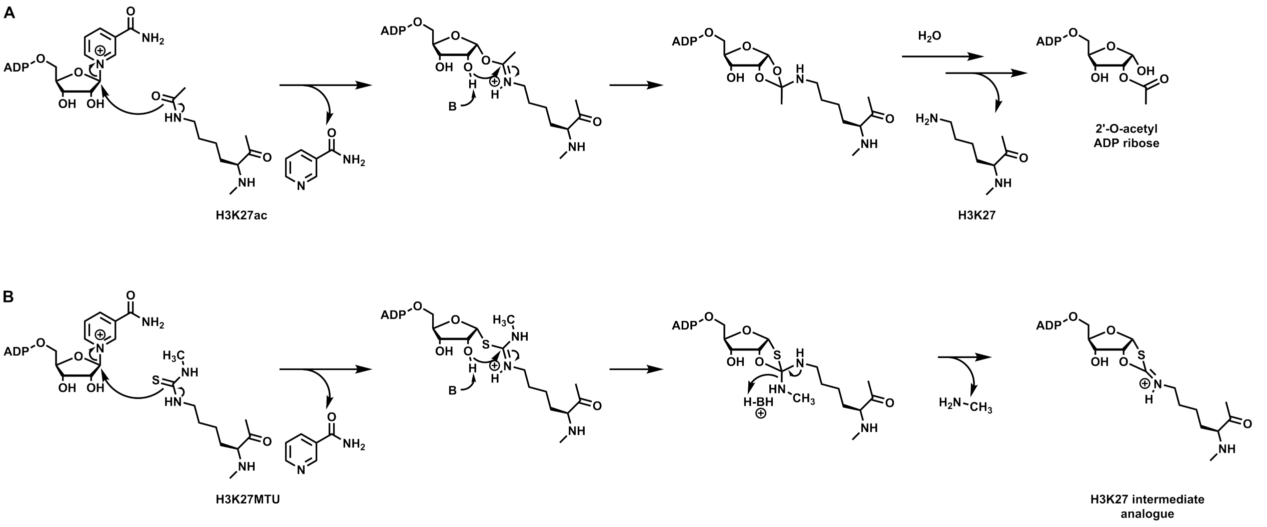

Figure S1. Mechanism of sirtuin NAD-dependent deacetylation and formation of covalent intermediate analogue. (A) Deacetylation mechanism. (B) Formation of N-methyl thiourea (MTU)

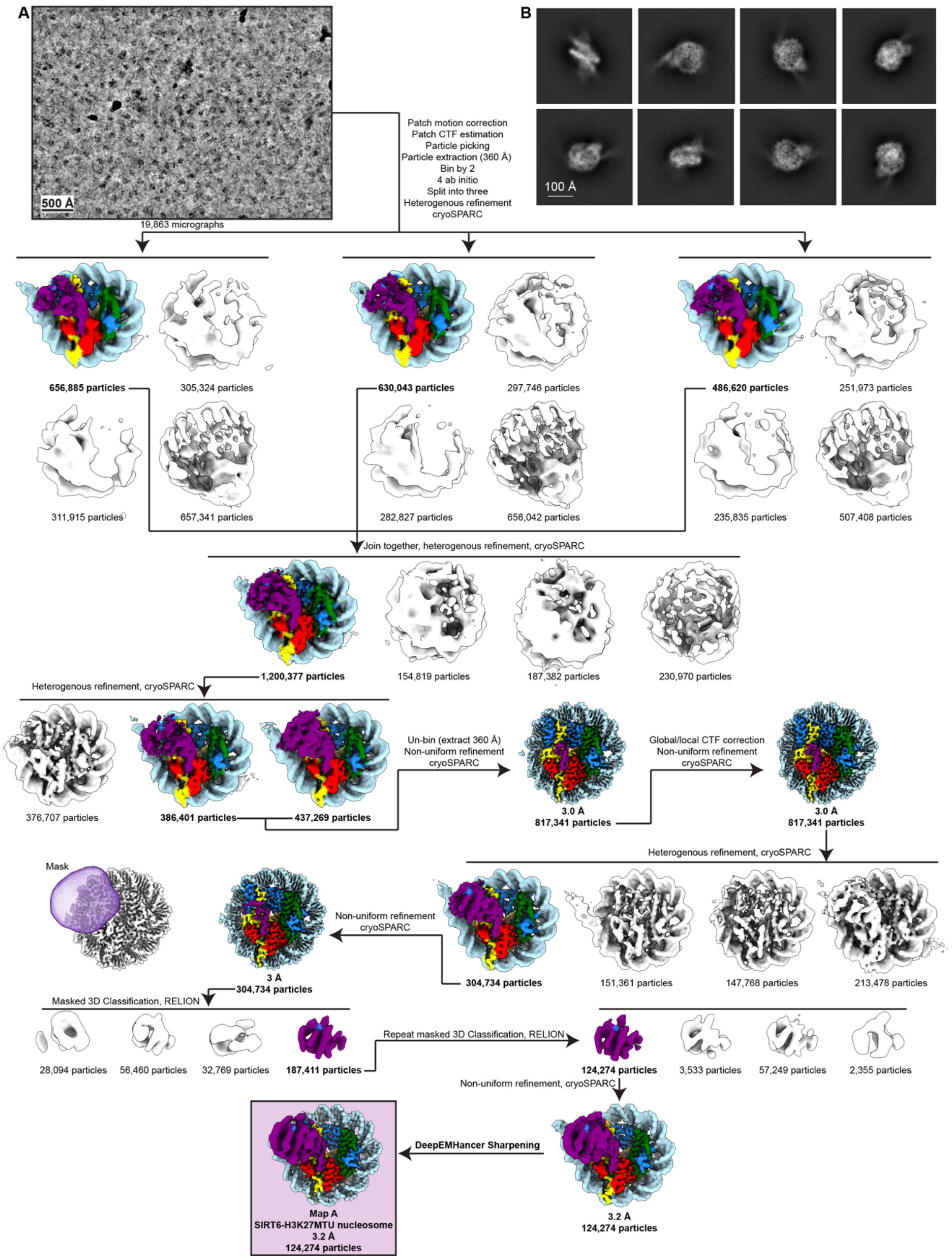

Figure S2. Data acquisition and processing of the SIRT6-H3K27MTU nucleosome complex. (A) Representative denoised micrograph with scale bar and subsequent cryo-EM processing. Final map is indicated. (B) 2D classes of final refinement with scale bar.

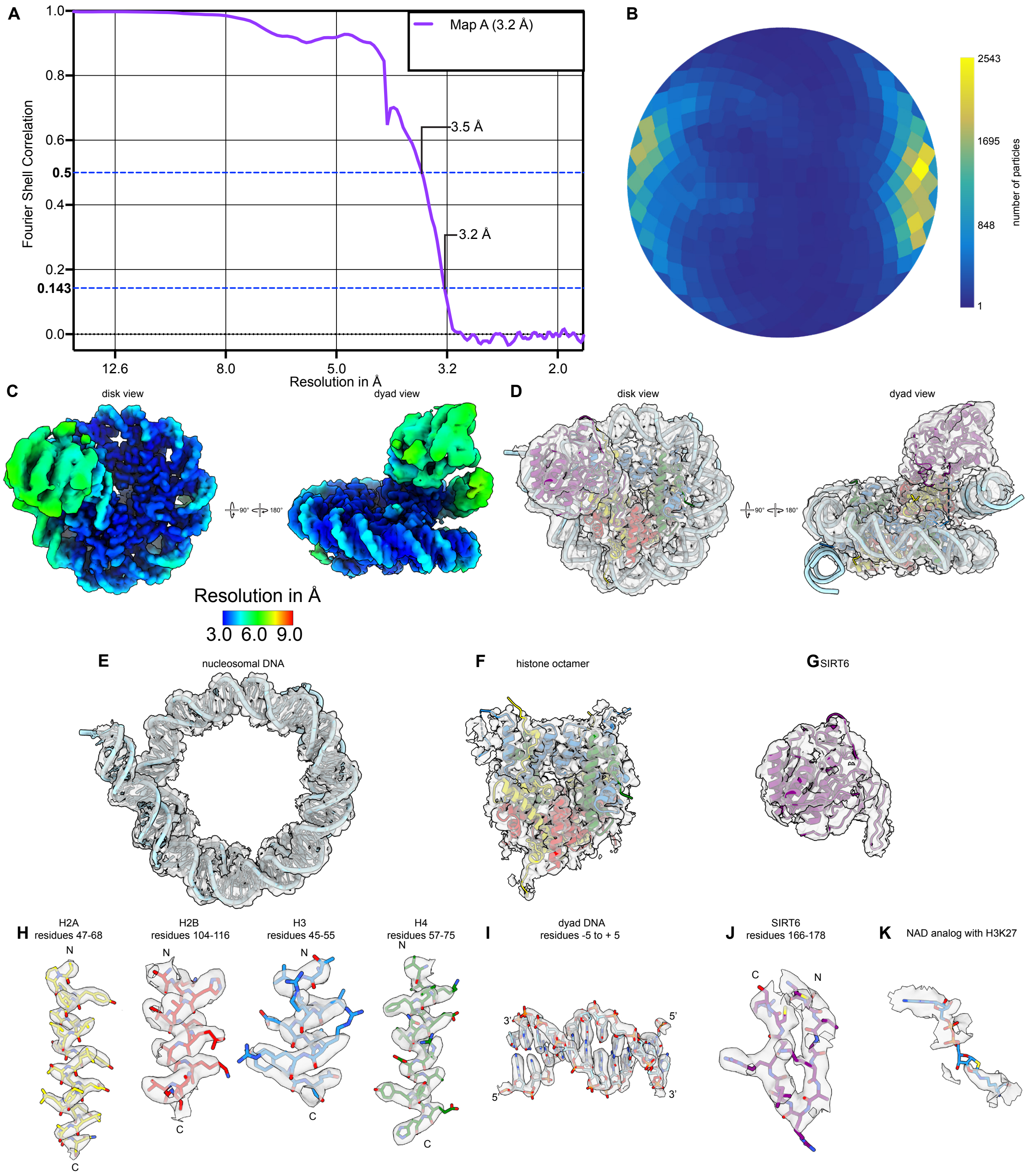

Figure S3. Cryo-EM Map and Model quality of SIRT6-H3K27MTU complex. (A) FSC curve of map A with 0.143 and 0.5 thresholds indicated. (B) Angular distribution of particles for map A. (C) Local resolution of map A. (D) Two views of SIRT6-H3K27MTU model with map A. (E) Coulomb potential map of DNA with fitted model (map A). (F) Coulomb potential map of histone octamer with fitted model (map A). (G) Coulomb potential map of SIRT6 with fitted model (map A). (H) Coulomb potential map of representative regions of histones H2A, H2B, H3, and H4 with fitted model (map A). (I) Coulomb potential map of representative region of DNA with fitted model (map A). (J) Coulomb potential map of representative region of SIRT6 with fitted model (map A). (K) Coulomb potential map of representative region of NAD analog with fitted model (map A).

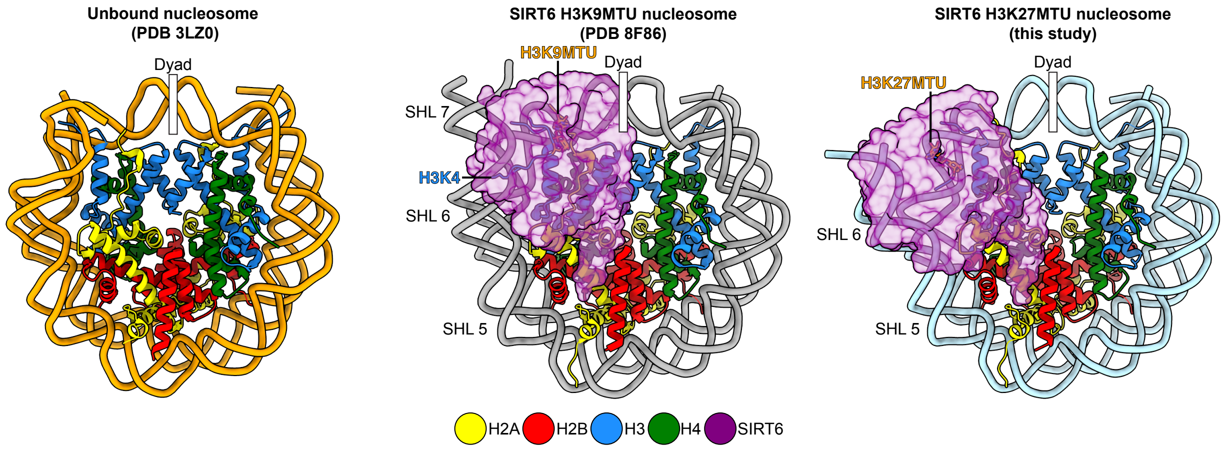

Figure S4. Comparison between unbound nucleosome, SIRT6 deacetylating H3K9, and SIRT6 deacetylating H3K27. H3K27 DNA (this study, light blue) is peeled off histone core starting at SHL 5. H3K9 DNA (PDB 8F86, dark gray) is slightly peeled off the histone core starting at SHL 5. Unbound nucleosome DNA (PDB 3LZ0, orange) is not peeled off.

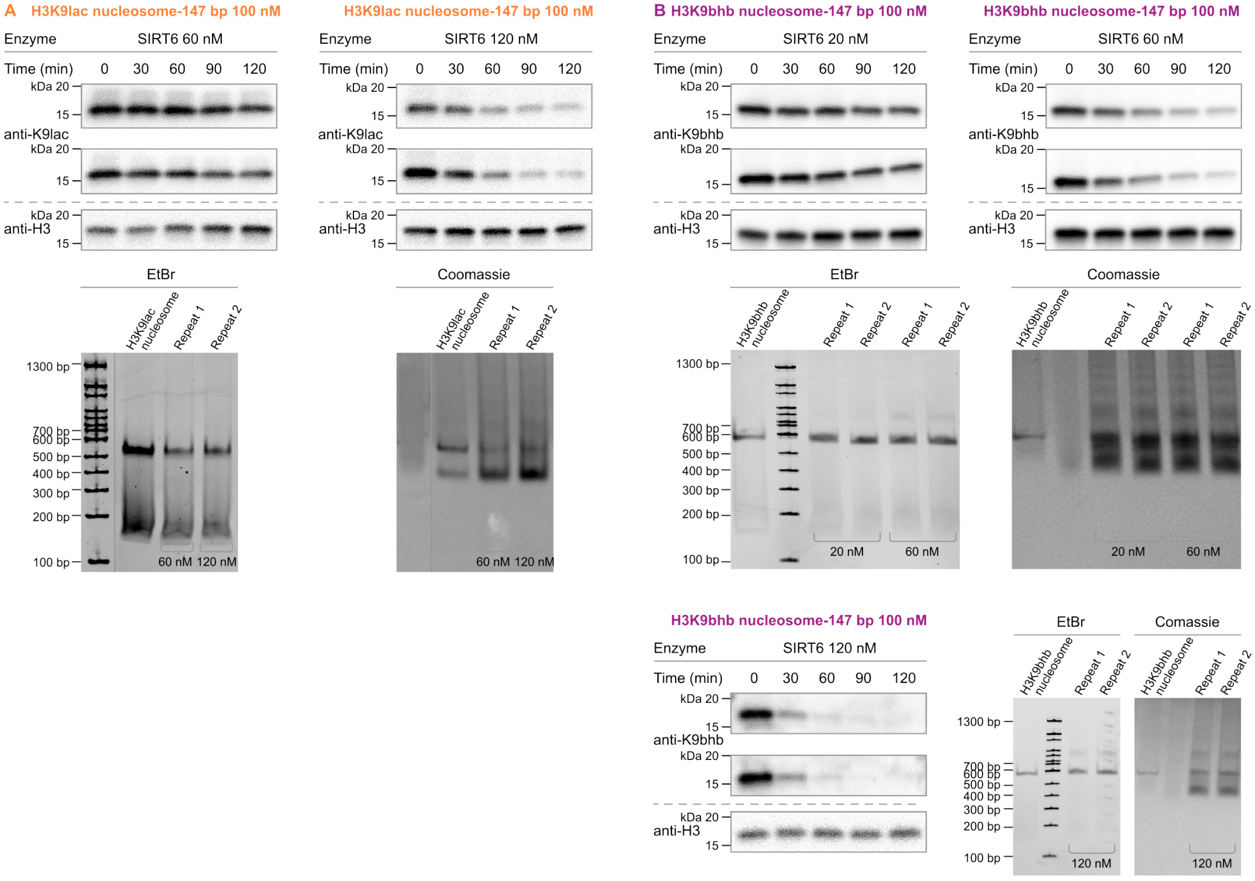

Figure S5. WT SIRT6 deacylation on H3K9 nucleosomes. Western blots and native gels of WT SIRT6 (2 different concentrations) deacylation assay on nucleosomes with (A) H3K9lac (n=4) (B) H3K9bhb (n=6).

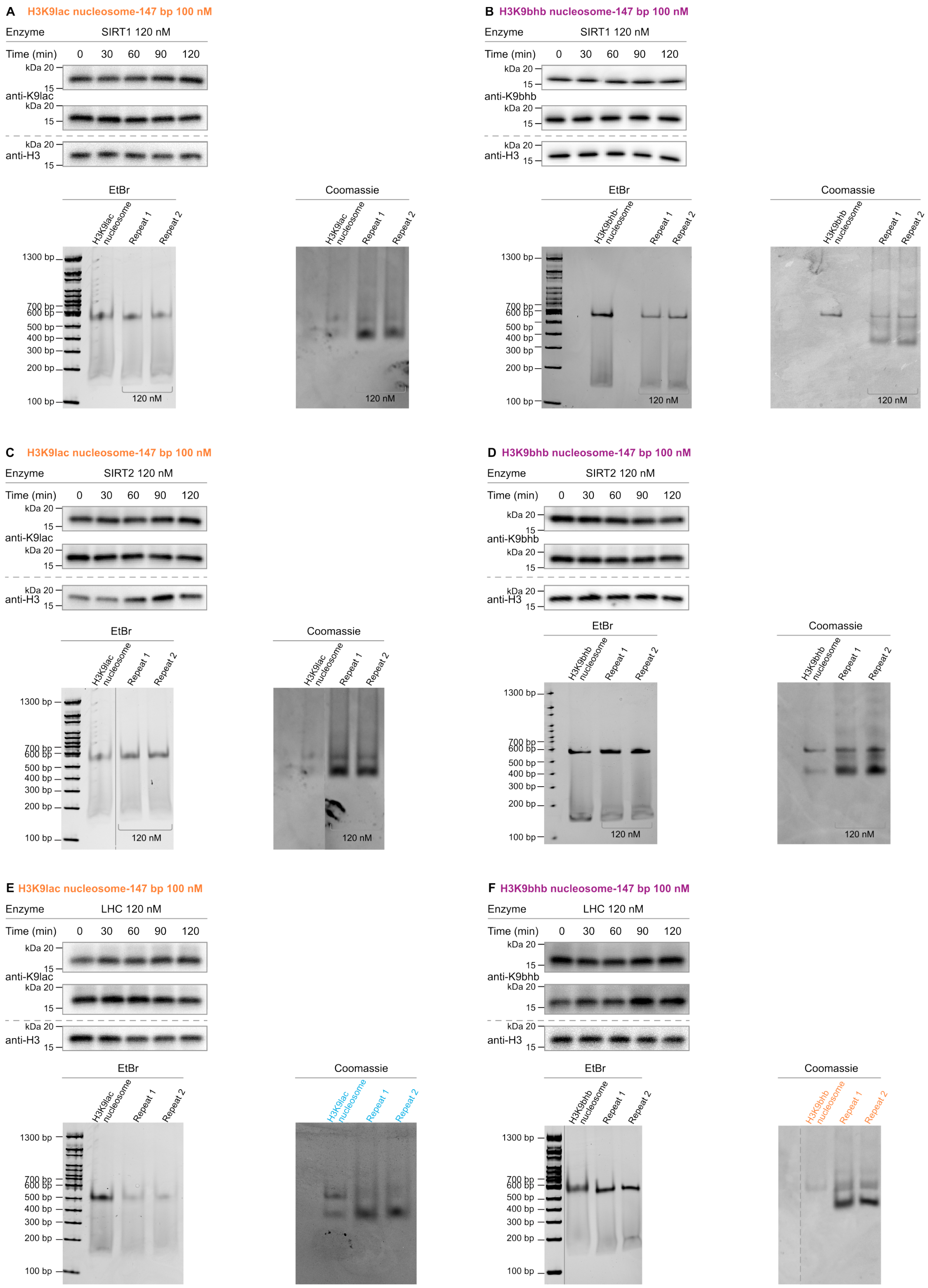

Figure S6. SIRT1, SIRT2, and LHC deacylation on H3K9 nucleosomes. Western blots and native gels of SIRT1 deacylation assay on nucleosomes with (A) H3K9lac (n=2), (B) H3K9bhb (n=2); SIRT2 on (C) H3K9lac (n=2), (D) H3K9bhb (n=2); LHC on (E) H3K9lac (n=2), (F) H3K9bhb (n=2).

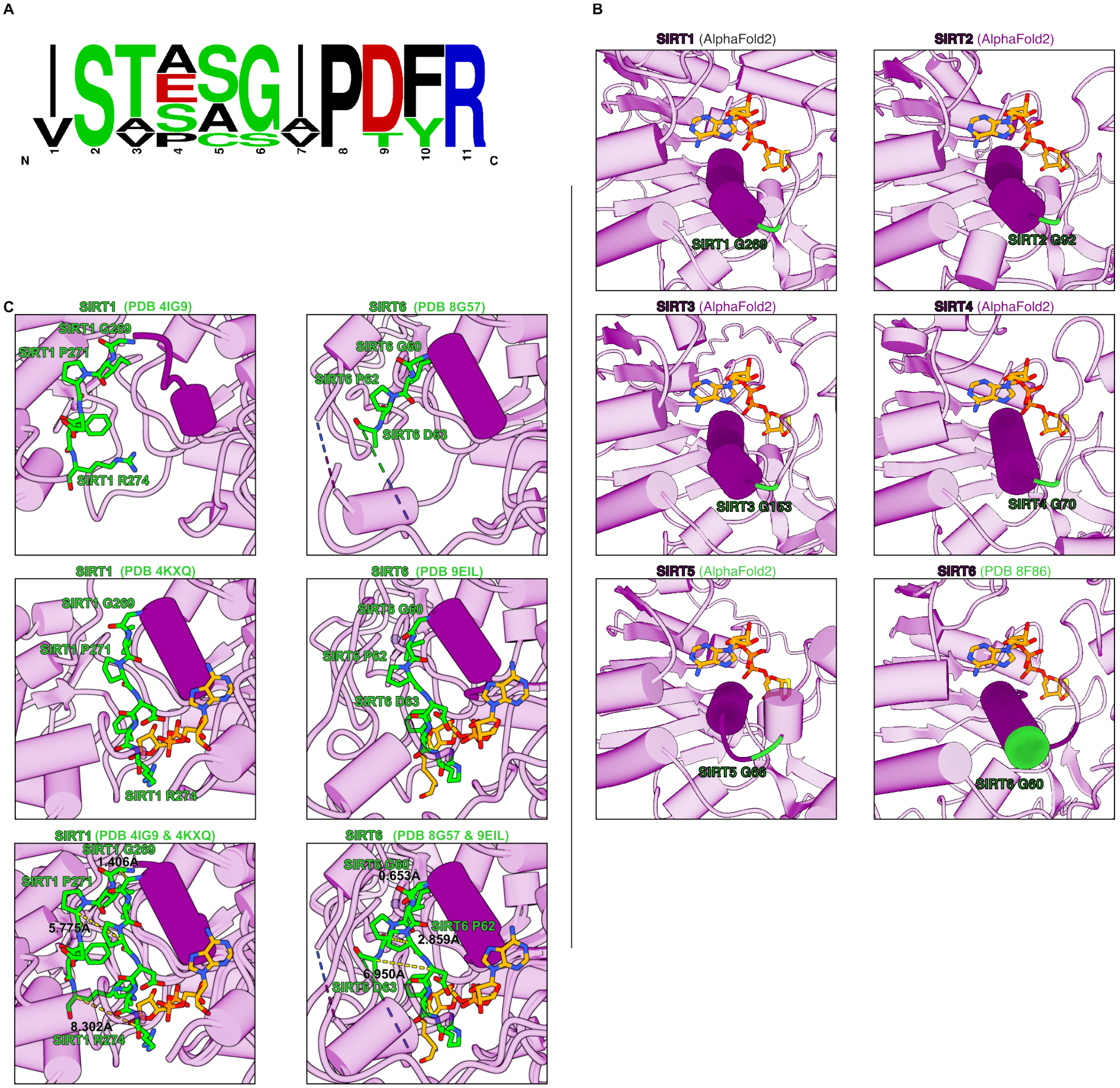

Figure S7. Alignment of G60 in SIRT6 with other sirtuins. (A) Sequence Logo representing the conserved amino acid sequence among human sirtuin enzymes. This logo displays the frequency of amino acids at each position, with the height of each letter indicating the degree of conservation. Common amino acids in the sequence are highlighted, illustrating the shared structural and functional motifs across sirtuin family members. (B) Structural models of sirtuin enzymes (SIRT1 to SIRT6) predicted using AlphaFold2 (except SIRT6, represented with PDB 8F86). Each panel shows the positioning of the conserved amino acid residue G in the Schellman motif capping the Rossman fold α2 helix, along with the location of NAD within the catalytic site. (C) Comparison of α2-α3 linker positions from published structures of two sirtuin (SIRT1 and SIRT6) apo (top) and holo (middle) forms. A composite structure (bottom) shows the distance moved by the glycine (SIRT1 G269, SIRT6 G60), proline (SIRT1 P271, SIRT6 P62) and arginine (SIRT1 R274) or aspartate (SIRT6 D63) of the α2-α3 linker.

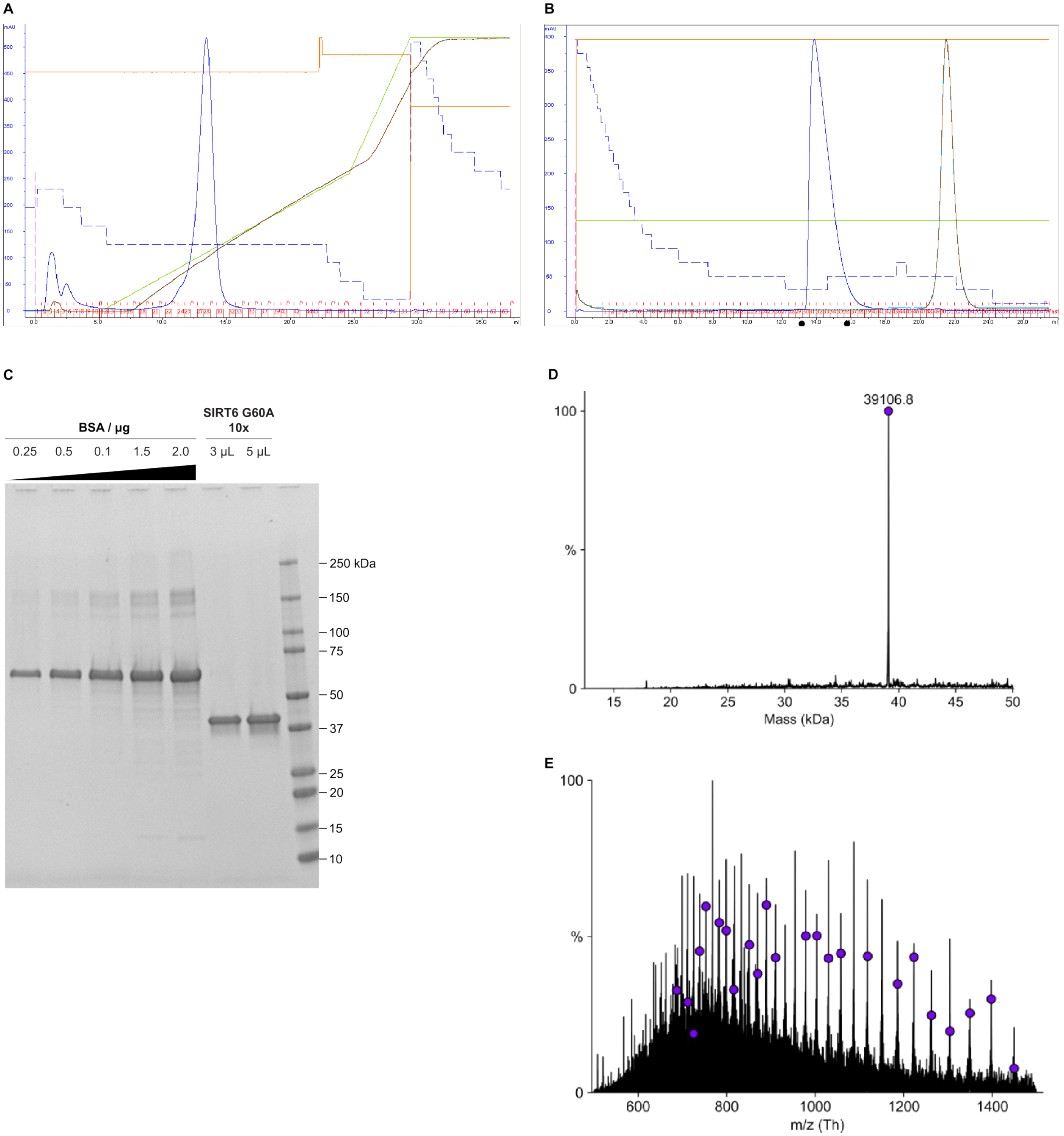

Figure S8. G60A SIRT6 purification and ESI characterization. (A) Heparin column FPLC chromatography for G60A SIRT6 purification. (B) Superdex200 column FPLC chromatography for G60A SIRT6 purification. (C) SDS-PAGE and Coomassie staining of final G60A SIRT6 after TEV cleavage. (D) Deconvoluted mass spectrum of G60A SIRT6 (purple circle) calculated $C_{1719}H_{2772}N_{514}O_{503}S_{13}$ $[M]^+$: 39104.84 Da, found: 39106.8 Da. (E) Raw mass spectrum of G60A SIRT6 (purple circle).

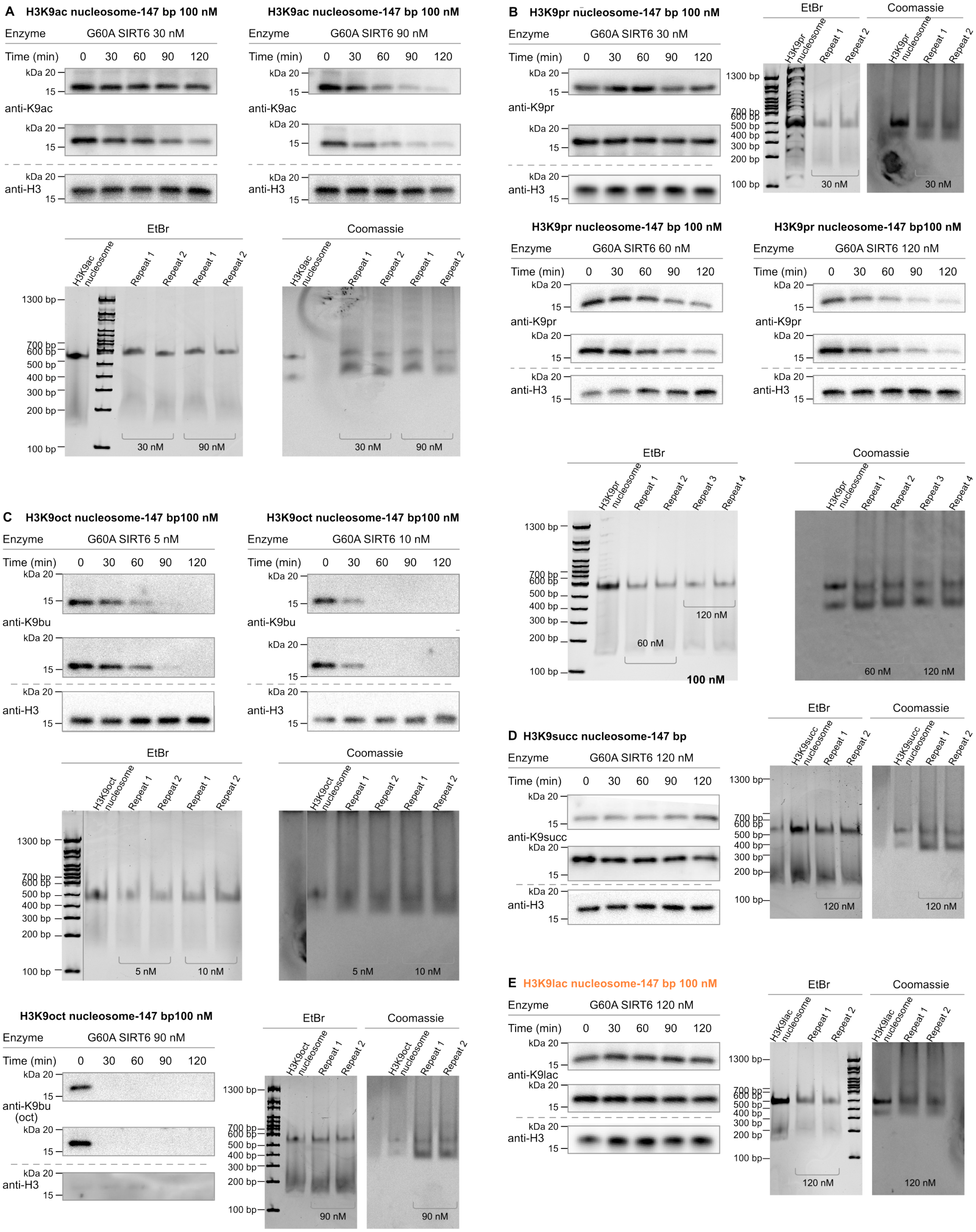
A
H3K9ac nucleosome-147 bp 100 nM
Enzyme G60A SIRT6 30 nM
Time (min) 0 30 60 90 120
anti-K9ac
anti-H3
H3K9ac nucleosome-147 bp 100 nM
Enzyme G60A SIRT6 90 nM
EtBr
Coomassie
H3K9ac nucleosome
Repeat 1
Repeat 2
30 nM
90 nM
B
H3K9pr nucleosome-147 bp 100 nM
Enzyme G60A SIRT6 30 nM
anti-K9pr
H3K9pr nucleosome
Enzyme G60A SIRT6 60 nM
H3K9pr nucleosome-147 bp100 nM
Enzyme G60A SIRT6 120 nM
Repeat 3
Repeat 4
60 nM
120 nM
100 nM
C
H3K9oct nucleosome-147 bp100 nM
Enzyme G60A SIRT6 5 nM
Enzyme G60A SIRT6 10 nM
anti-K9bu
H3K9oct nucleosome
5 nM
10 nM
Enzyme G60A SIRT6 90 nM
anti-K9bu (oct)
90 nM
D
H3K9succ nucleosome-147 bp
Enzyme G60A SIRT6 120 nM
anti-K9succ
H3K9succ nucleosome
E
H3K9lac nucleosome-147 bp 100 nM
anti-K9lac
H3K9lac nucleosome
kDa 20
15
1300 bp
700 bp
600 bp
500 bp
400 bp
300 bp
200 bp
100 bp

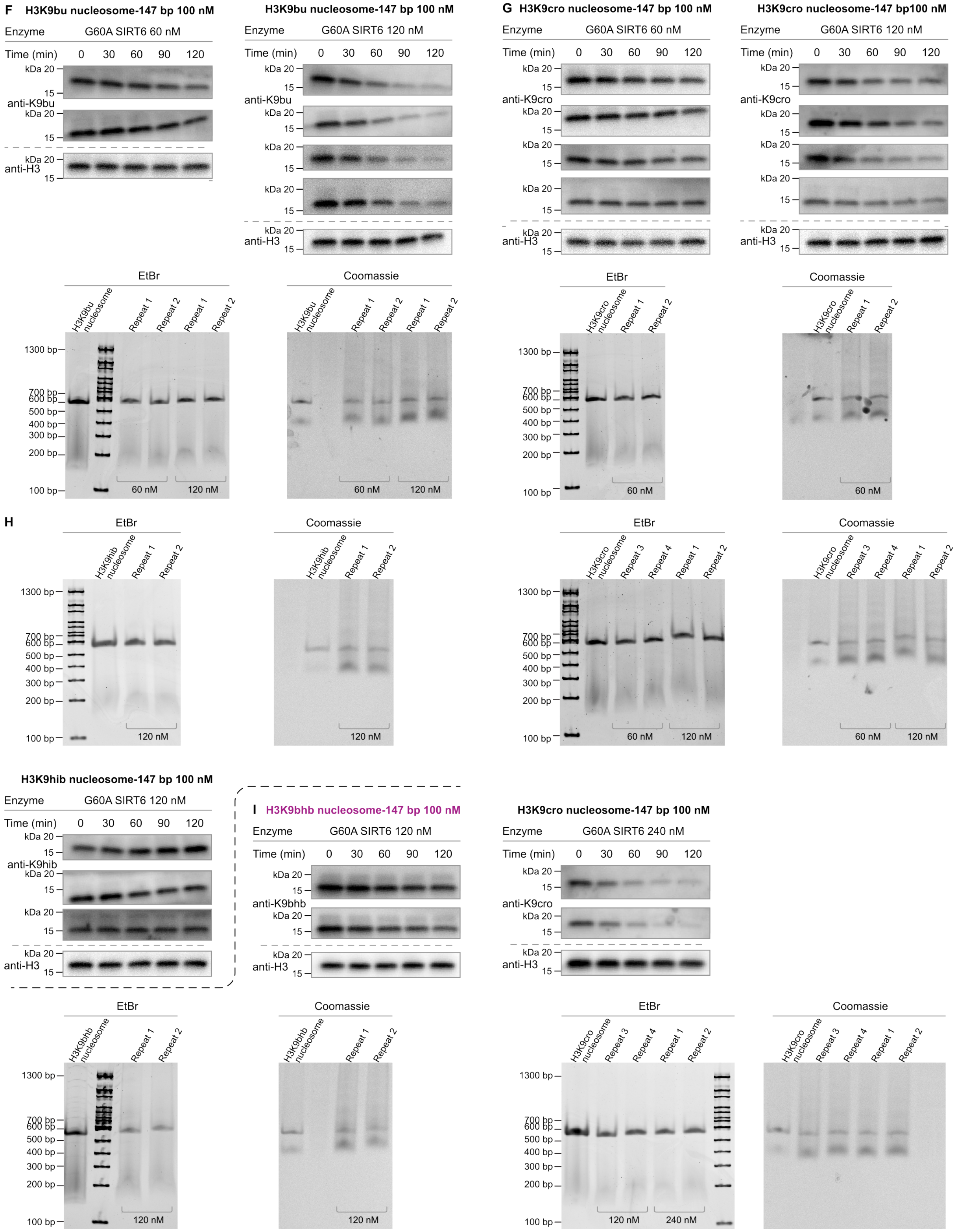

Figure S9. G60A SIRT6 deacylation on H3K9 nucleosomes. Western blots and native gels of G60A SIRT6 (1 or 2 or 3 different concentrations) deacylation assay on nucleosomes with (A) H3K9ac (n=4), (B) H3K9pr (n=6), (C) H3K9oct (n=6), (D) H3K9succ (n=2), (E) H3K9lac (n=2), (F) H3K9bu (n=6), (G) H3K9cro (n=10), (H) H3K9hib (n=3), (I) H3K9bhb (n=2).

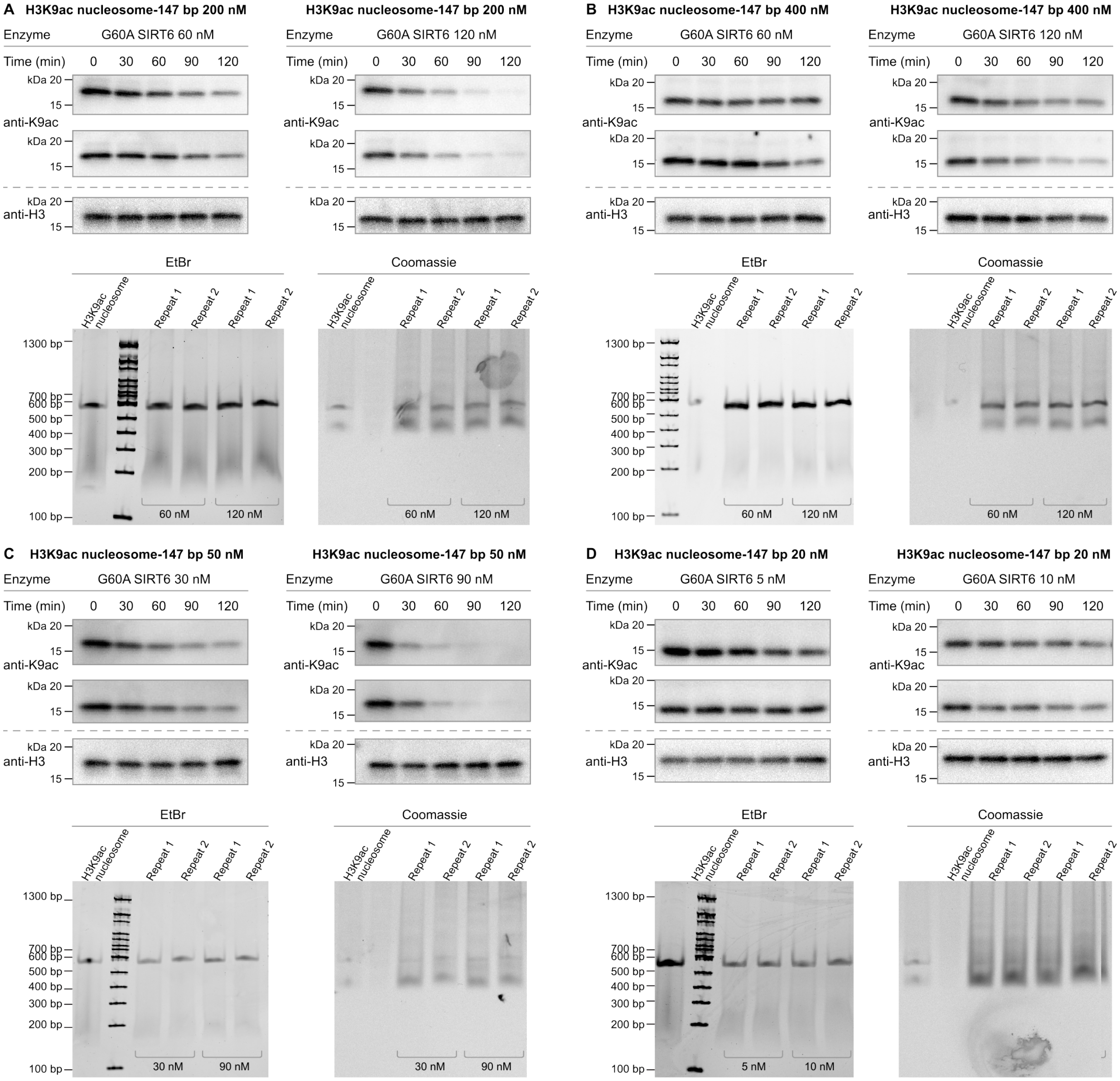

E

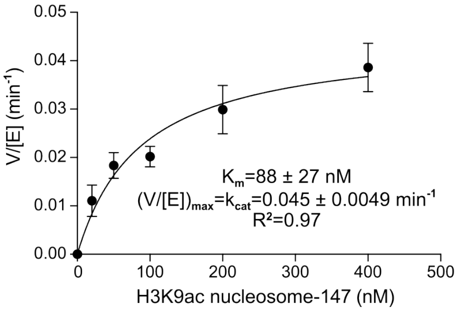

F

Nucleosome-147 (nM)	$V/[E](min^{-1})$
0	
20	0.011 ± 0.0032
50	0.018 ± 0.0026
100	0.020 ± 0.0019
200	0.030 ± 0.0050
4000	0.039 ± 0.0048

Figure S10. Km of G60A SIRT6 for H3K9ac nucleosome-147. Western blots and native gels of G60A SIRT6 (2 different concentrations) deacetylation assay on H3K9ac nucleosome-147 with nucleosome final concentration as (A) 200 nM (n=4), (B) 400 nM (n=4), (C) 50 nM (n=4), (D) 20 nM (n=4). (E) Michaelis–Menten curve fitting for V/[E] at different nucleosome concentrations. (F) Table for H3K9ac nucleosome kinetics with different nucleosome final concentration. Kinetic values shown are ± SD.

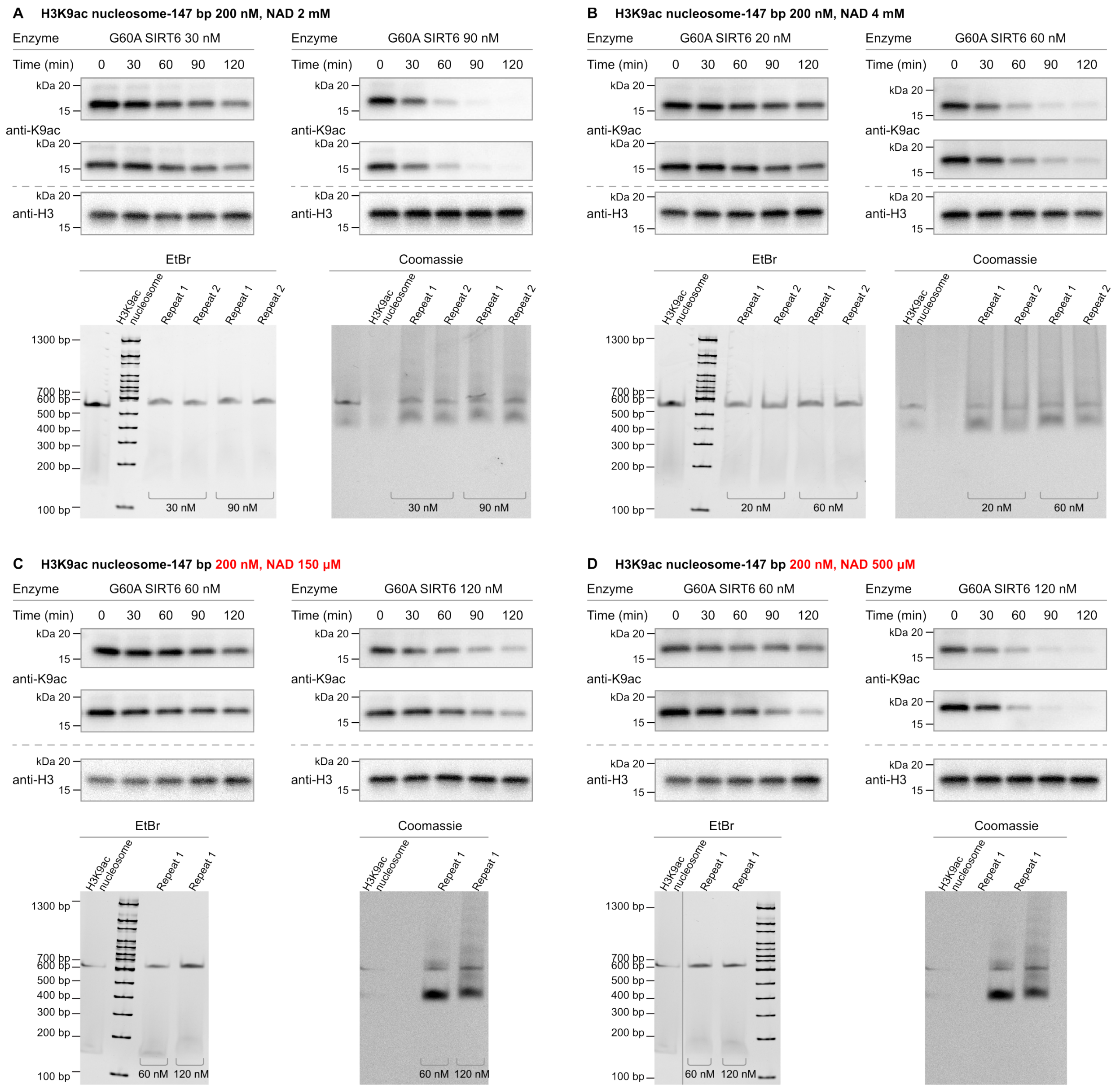

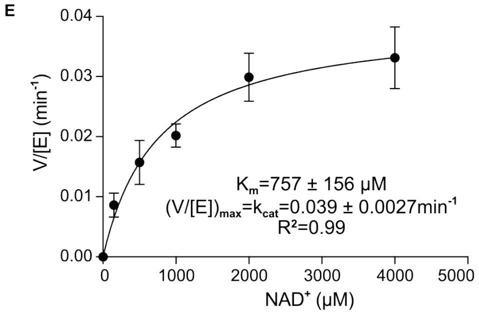

F

NAD (μM)	V/[E](min^{-1})
0	
150	0.0086 ± 0.0020
500	0.016 ± 0.0036
1000	0.020 ± 0.0019
2000	0.030 ± 0.0040
40000	0.033 ± 0.0050

Figure S11. Km of G60A SIRT6 for NAD. Western blots and native gels of G60A SIRT6 (10 nM and 30 nM) deacetylation assay on H3K9ac nucleosome-147 with (A) 2 mM (n=4), (B) 4 mM (n=4), (C) 150 μM NAD (n=4), (D) 500 μM NAD (n=4). (E) Michaelis–Menten curve fitting for V/[E] with different concentration of NAD. (F) Table for H3K9ac nucleosome kinetic parameters with different concentration of NAD. Kinetic values shown are ± SD.

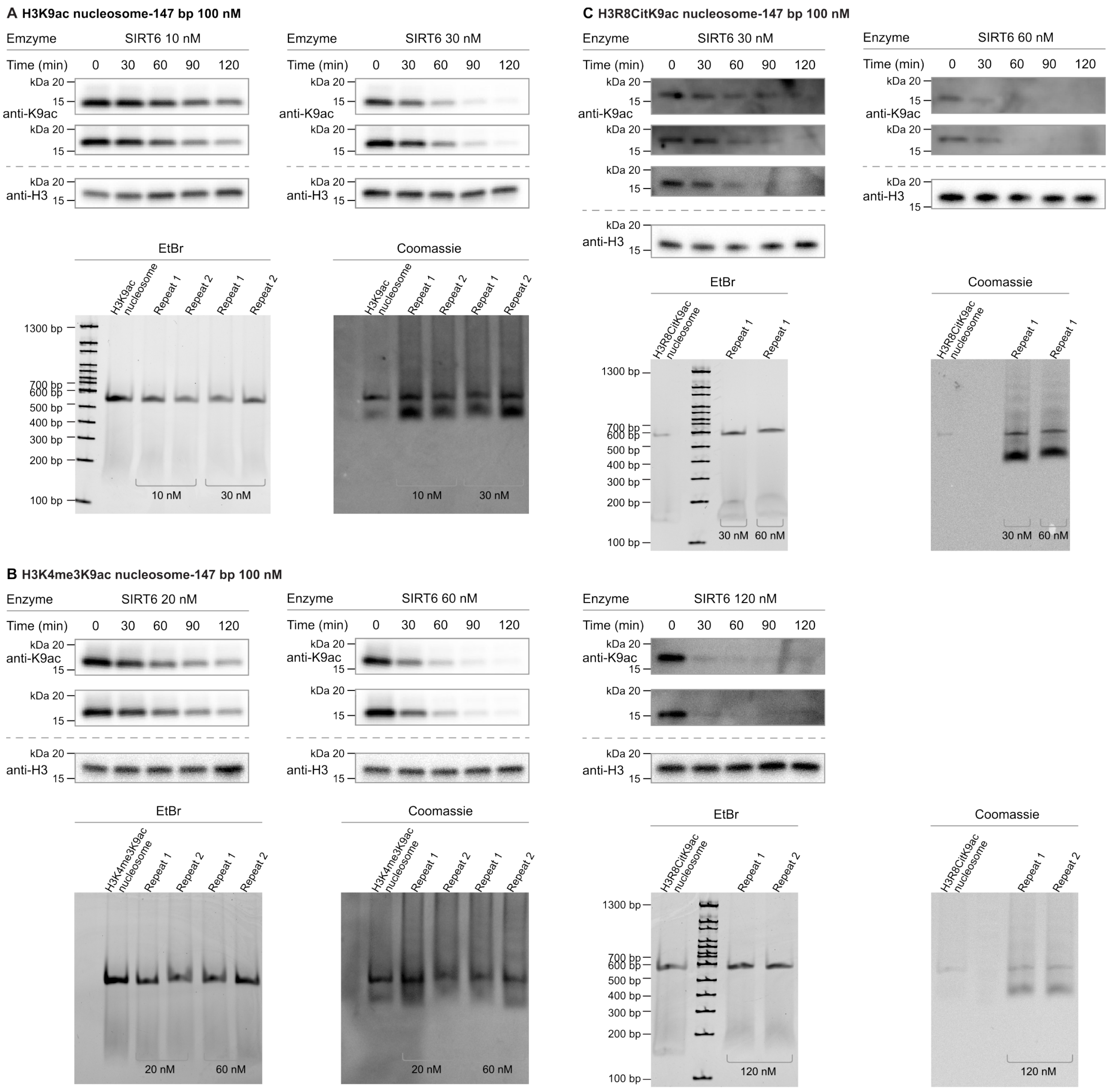

Figure S12. SIRT6 deacylation of nucleosomes with H3K9ac and proximal modifications. Western blots and native gels of WT SIRT6 (2 or 3 different concentrations) deacylation assay with nucleosomes (A) H3K9ac (n=4), (B) H3K4me3K9ac (n=4), (C) H3R8CitK9ac (n=7).

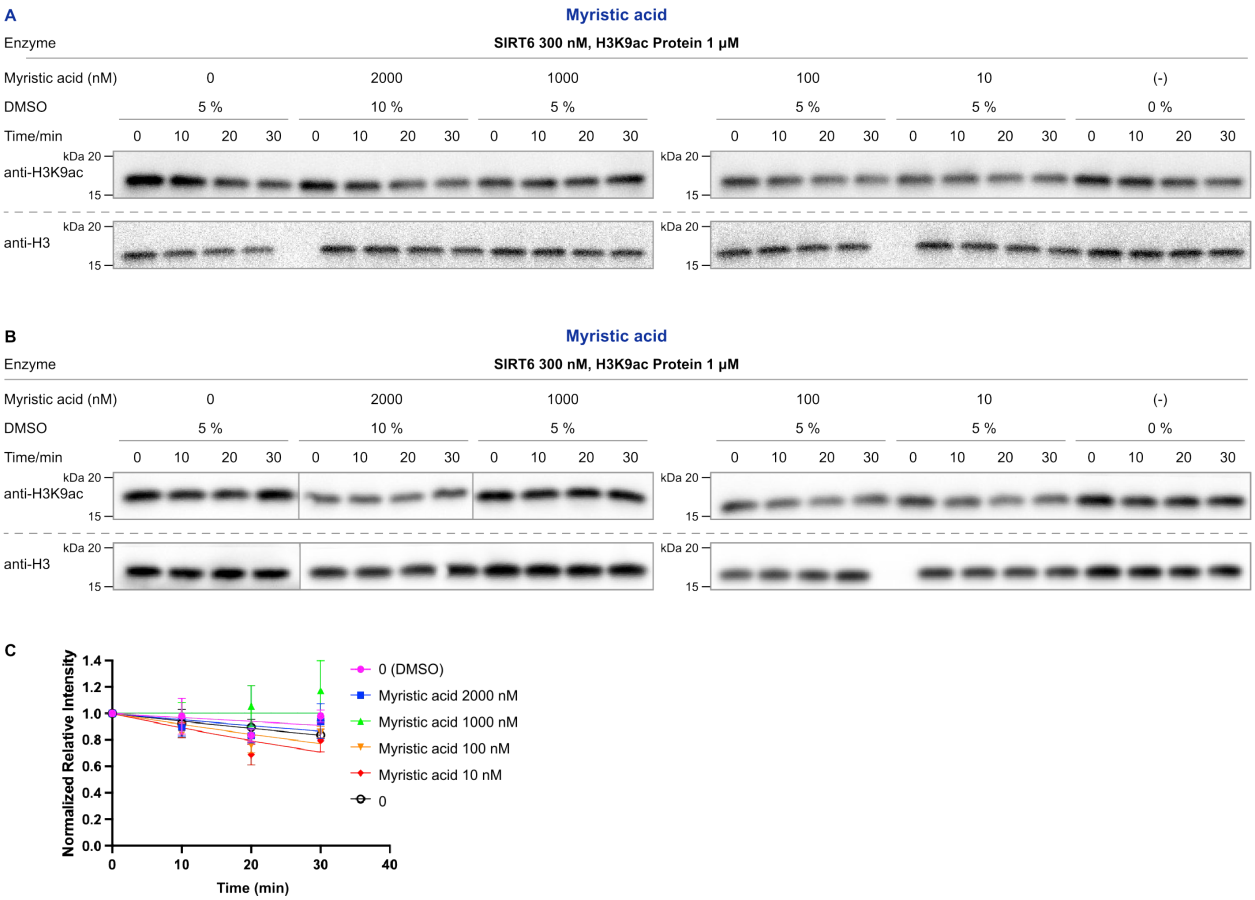

Figure S13. Myristic acid effects on Sirt6 activity on H3K9ac free histone H3 protein. Western blots and native gels of 300 nM SIRT6 WT deacetylation assay on 1 μM H3K9ac protein with (A-B) different concentrations of myristic acid in 5-10 % DMSO, 5 % DMSO vehicle alone, or no DMSO (n=2). (C) Curve fitting for H3K9ac protein kinetics with different myrstic acid concentrations. Kinetic values shown are ± SD.

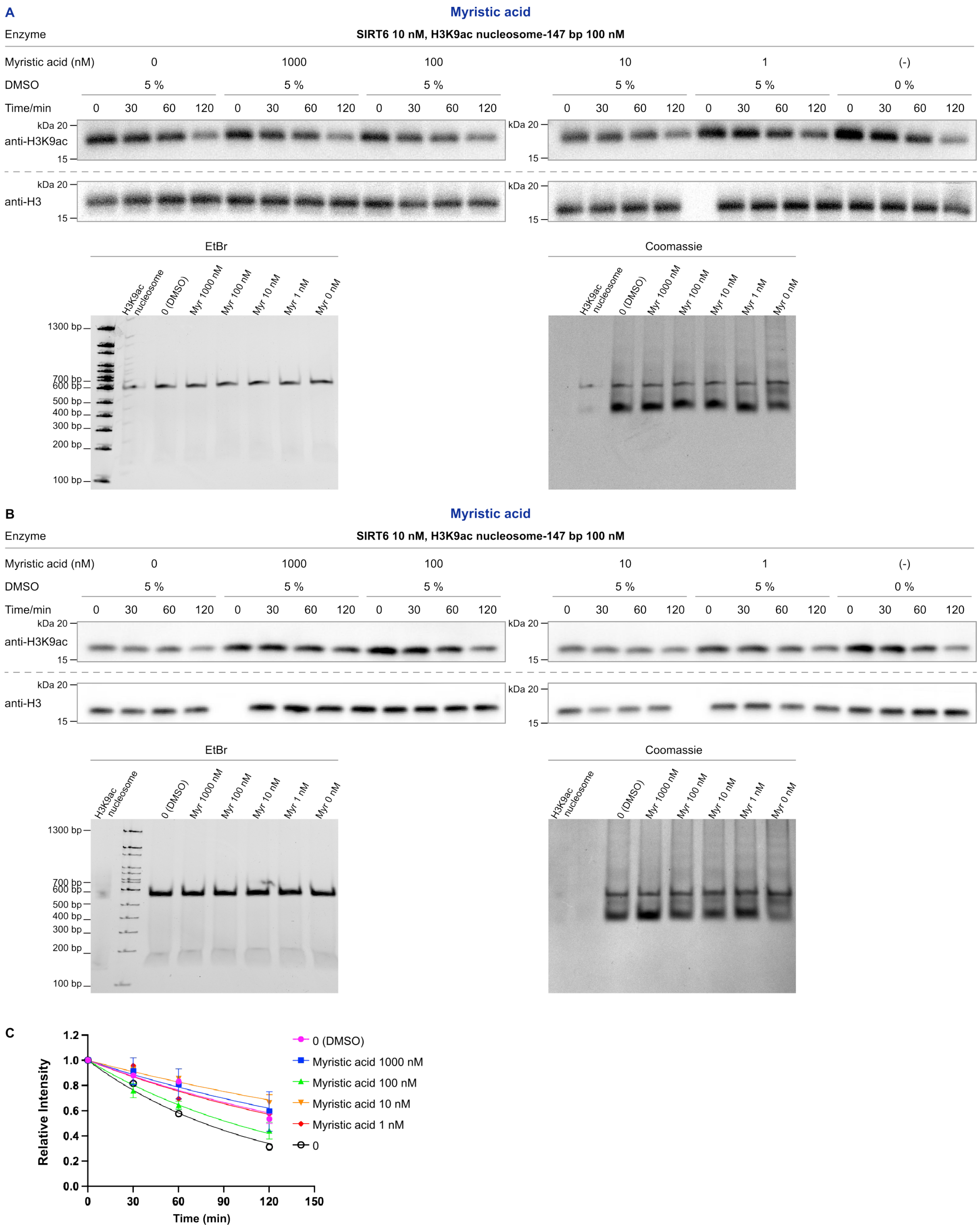

Figure S14. Myristic acid effects on Sirt6 activity on H3K9ac nucleosome-147, Trial 1. Western blots and native gels of 10 nM SIRT6 WT deacetylation assay on 100 nM H3K9ac nucleosome-147 with (A-B) different concentrations of myristic acid in 5 % DMSO, DMSO vehicle alone, or no DMSO (n=2). (C) Curve fitting for H3K9ac nucleosome kinetics with different myristic acid concentrations. Kinetic values shown are ± SD.

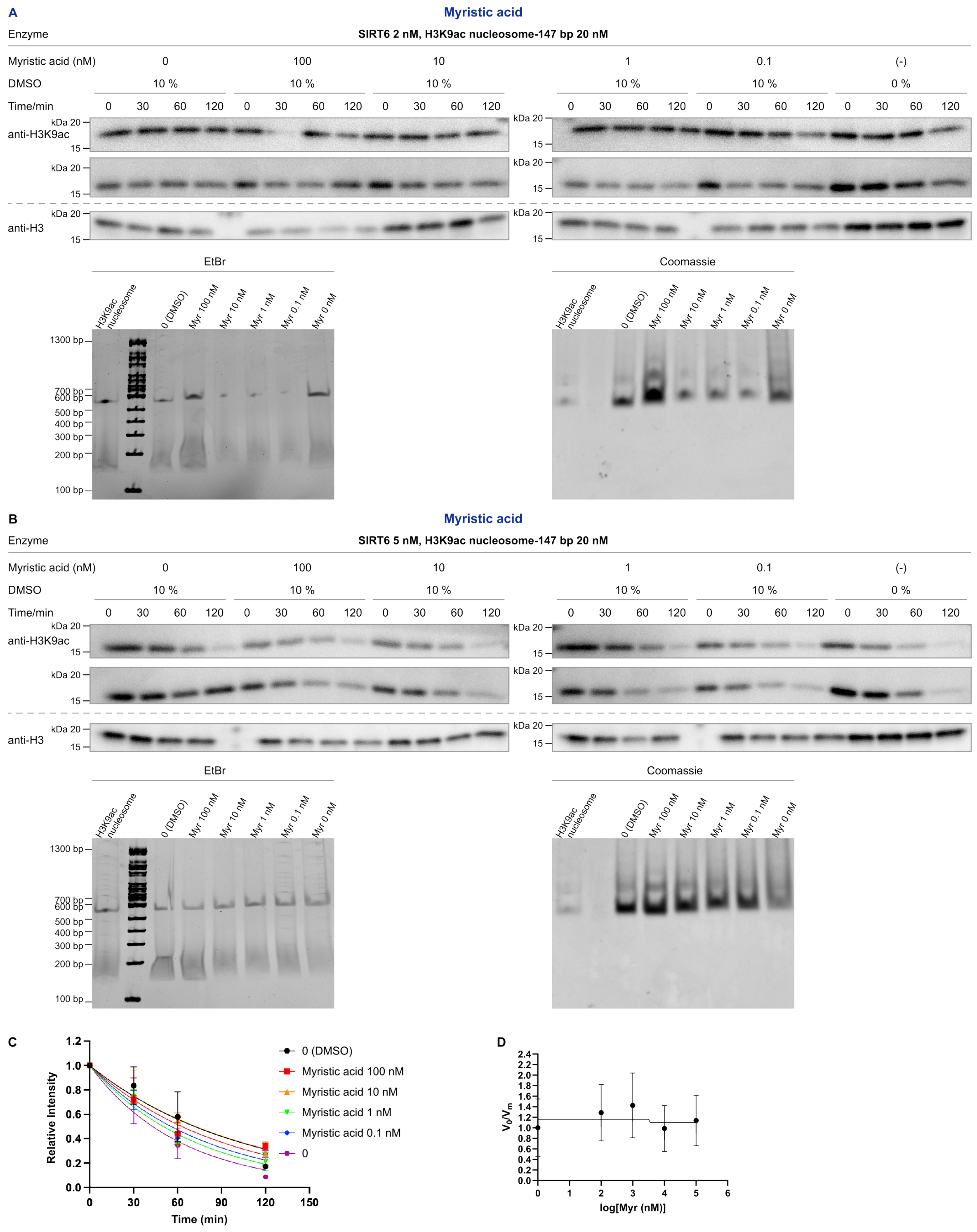

Figure S15. Myristic acid effects on Sirt6 activity on H3K9ac nucleosome-147, Trial 2. Western blots and native gels of 5 nM SIRT6 WT deacetylation assay on 20 nM H3K9ac nucleosome-147 with (A-B) different concentrations of myristic acid in 10 % DMSO, DMSO vehicle alone, or no DMSO (n=4). (C) Curve fitting for H3K9ac nucleosome kinetics with different myristic acid concentrations. (D) Curve fitting of "log(activator) vs. response -- Variable slope (four parameters)" with logIC50(nM) = ~ 3.5 (IC50 = ~3.4 μM), Hill slope = -56, suggesting almost no effect. Kinetic values shown are ± SD.

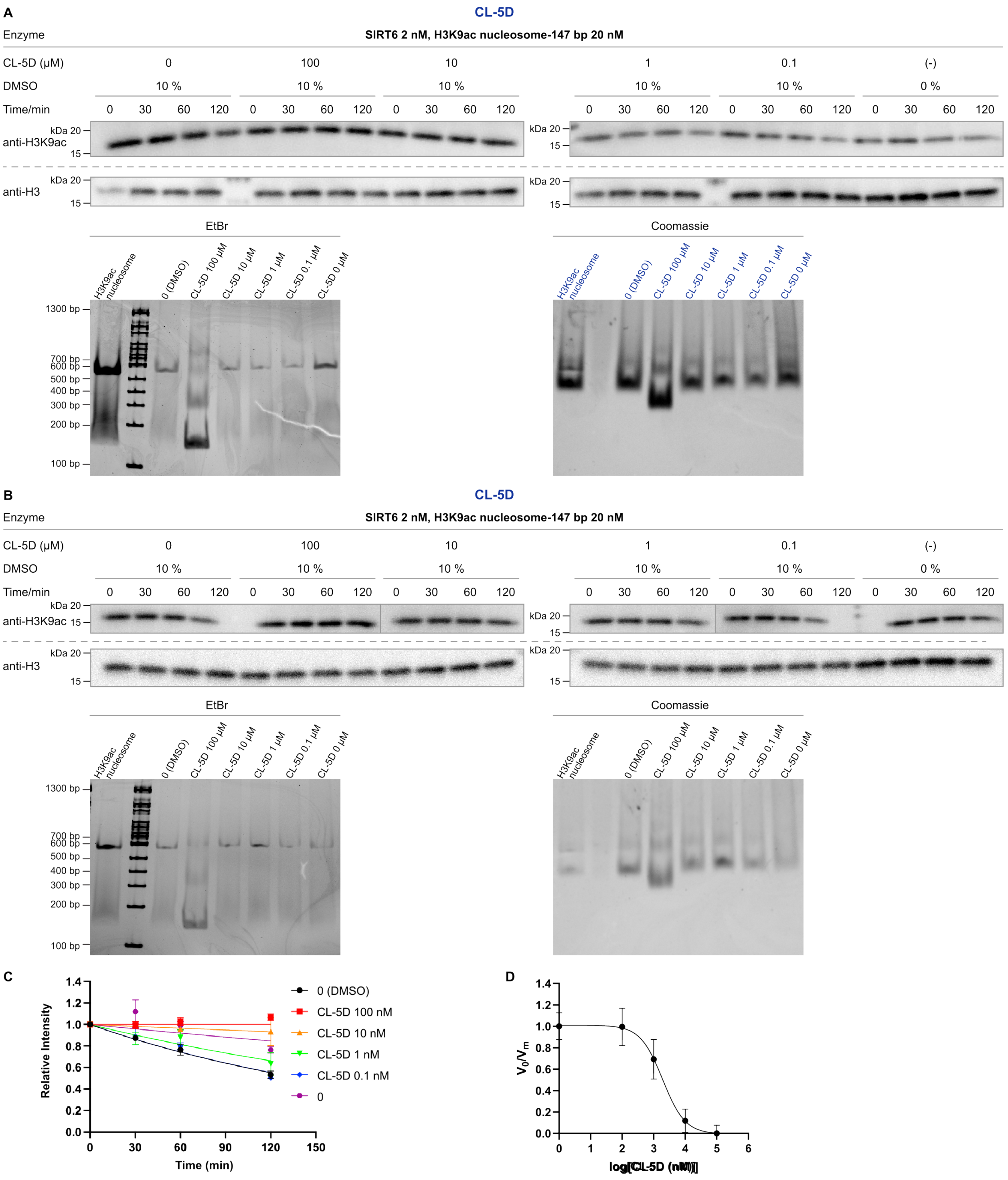

Figure S16. CL-5D effects on Sirt6 activity with H3K9ac nucleosome-147, Trial 1. Western blots and native gels of 2 nM WT SIRT6 deacetylation assay with 20 nM H3K9ac nucleosome-147 with (A-B) different concentrations of CL-5D in 10 % DMSO, DMSO vehicle alone, or no DMSO (n=2). (C) Curve fitting for H3K9ac nucleosome kinetics with different CL-5D concentrations. (D) Curve fitting of "log(activator) vs. response -- Variable slope (four parameters)" with logIC50(nM) = 3.3±0.037 (IC50 = ~1.9±0.18 μM), Hill slope = -1.2. Kinetic values shown are ± SD.

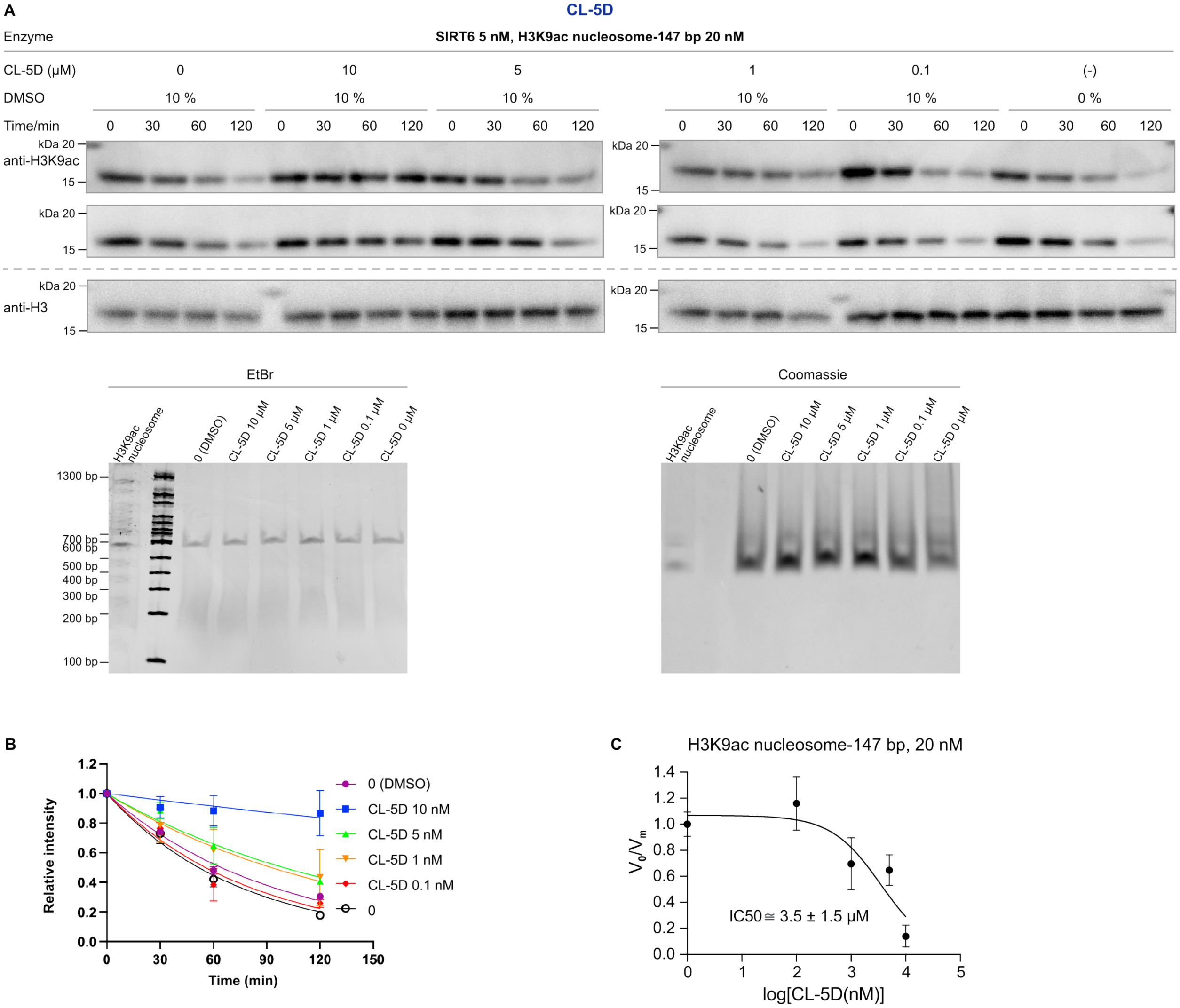

Figure S17. CL-5D effects on Sirt6 activity with H3K9ac nucleosome-147, Trial 2. Western blots and native gels of 5 nM SIRT6 WT deacetylation assay with 20 nM H3K9ac nucleosome-147 with (A) different concentrations of CL-5D in 10 % DMSO, DMSO vehicle alone, or no DMSO (n=2). (B) Curve fitting for H3K9ac nucleosome kinetics with different CL-5D concentrations. (C) Curve fitting of "log(activator) vs. response -- Variable slope (four parameters)" with logIC50(nM) = 3.6±0.34 (IC50 = ~3.5±2.2 μM), Hill slope = 0.73. This panel is adapted from Figure 6F. Kinetic values shown are ± SD.

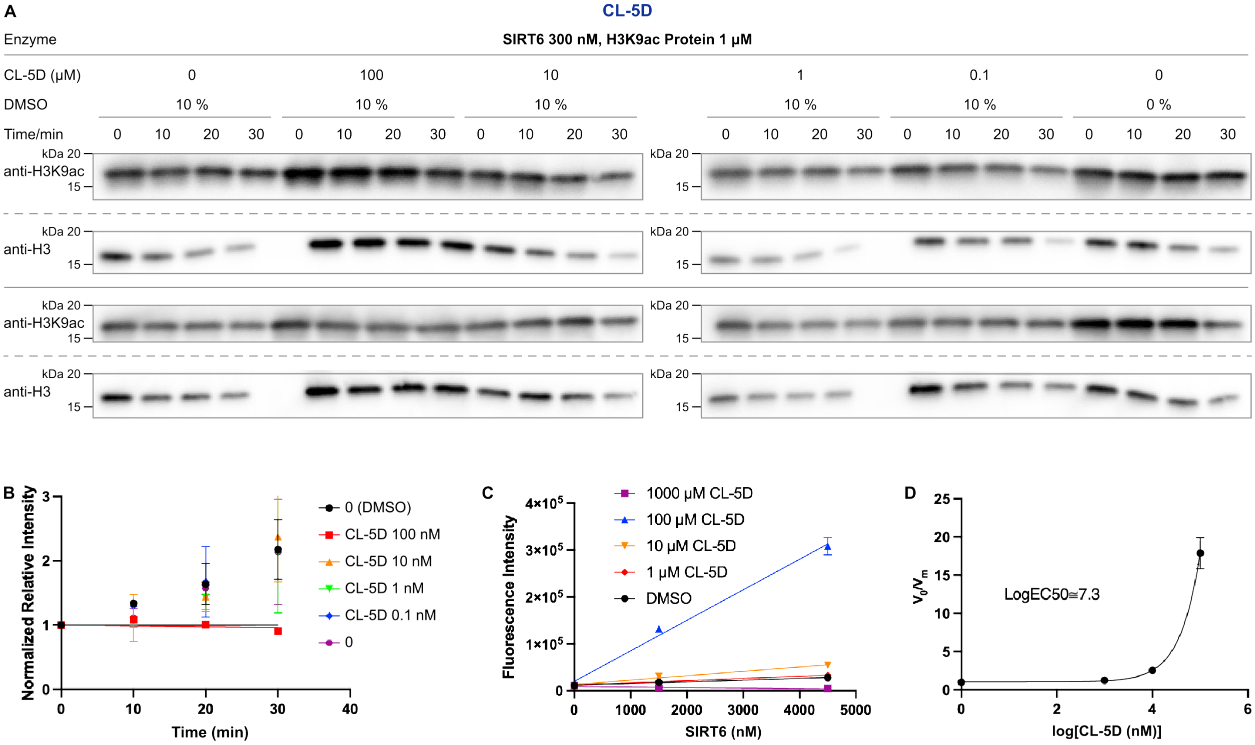

Figure S18. CL-5D effects on Sirt6 activity with H3K9ac free H3 protein or H3 tail peptide. Western blots and native gels of 300 nM SIRT6 WT deacetylation assay with 1 μM H3K9ac free histone H3 protein with (A) different concentrations of CL-5D in 10 % DMSO, DMSO vehicle alone, or no DMSO (n=2). (B) Curve fitting for H3K9ac protein kinetics with different CL-5D concentrations. (C) Linear fitting of Fluor de Lys assay of SIRT6 WT on H3K9ac peptide kinetic parameters at different CL-5D concentrations. (D) Curve fitting of Fluor de Lys assay with "log(activator) vs. response -- Variable slope (four parameters)" with logEC50(nM) = ~7.3 (EC50 = ~20 mM), Hill slope = 1.0. Kinetic values shown are ± SD.

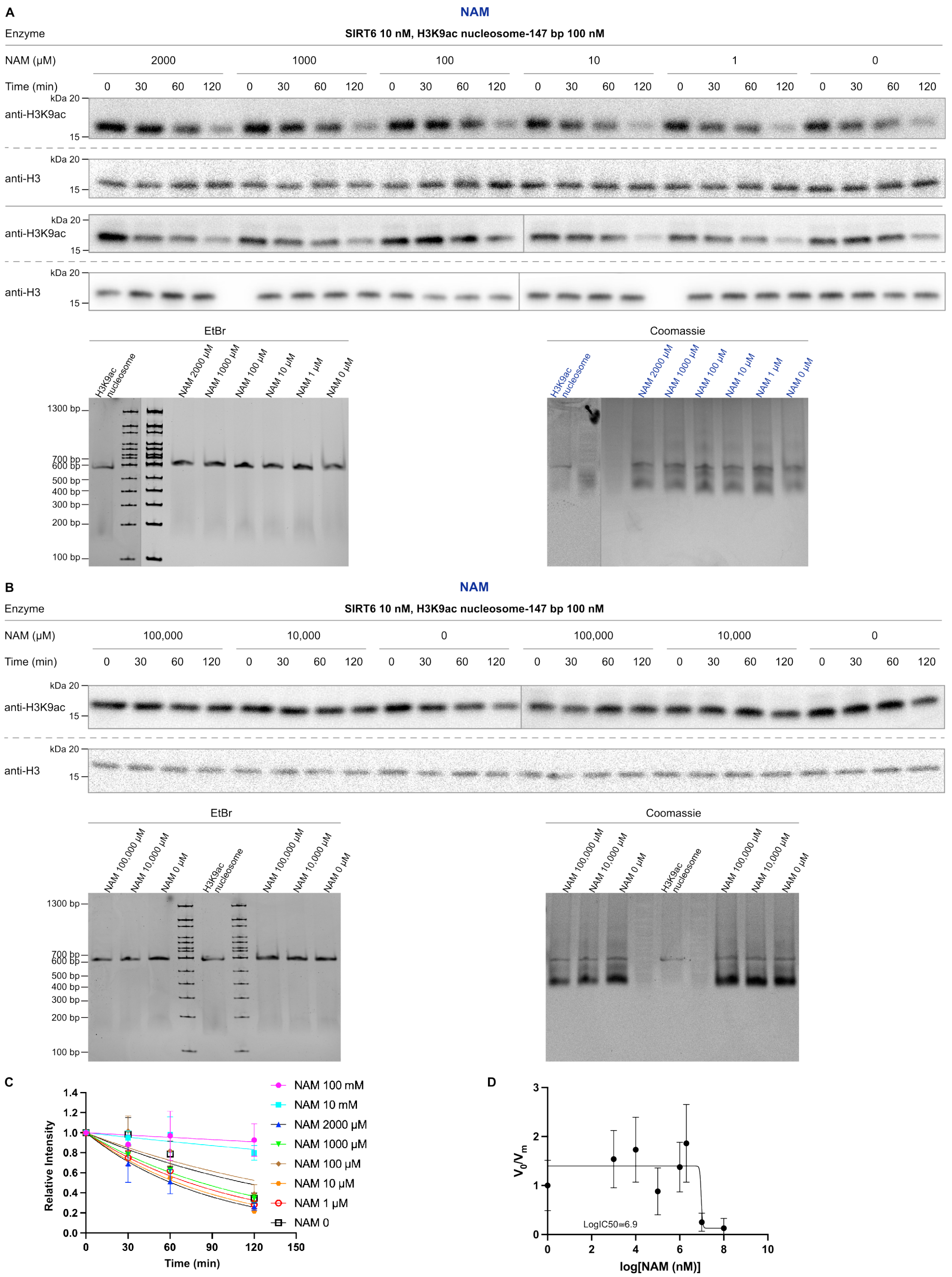

Figure S19. Nicotinamide (NAM) inhibition assay on H3K9ac nucleosome-147. Western blots and native gels of 10 nM SIRT6 WT deacetylation assay with 100 nM H3K9ac nucleosome-147 with (A-B) different concentrations of NAM or no NAM (n=2). (C) Curve fitting for H3K9ac nucleosome kinetics with different NAM concentrations. (D) Curve fitting of "log(activator) vs. response -- Variable slope (four parameters)" with logIC50(nM) = ~6.9 (IC50 = ~8.5 mM), Hill slope = -14. Kinetic values shown are ± SD.

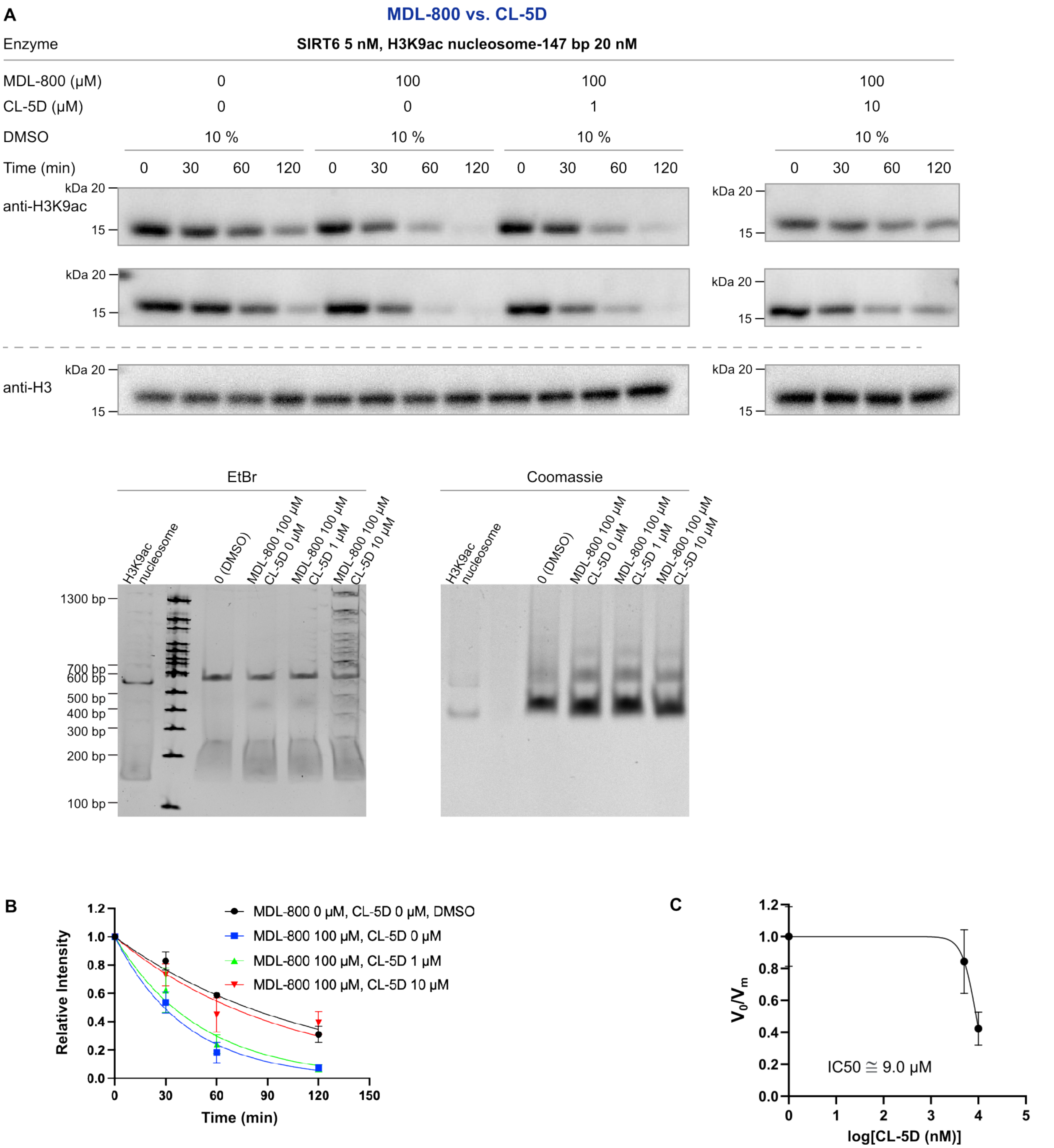

Figure S20. SIRT6 activity regulation by two small molecules. (A) Western blots and native gels analysis of the effect of CL-5D on SIRT6 WT deacetylation of H3K9ac 147 bp nucleosome substrates with 100 µM MDL-800 with DMSO as the vehicle control, as MDL-800 caused a significant rate increase alone compared with DMSO (n=2). (B) Fitting SIRT6 deacetylation rates at different concentrations of CL-5D under 100 µM MDL-800. (C) CL-5D activation curve fitting (n=2) under 100 µM MDL-800 of "log(activator) vs. response -- Variable slope (four parameters)" with logIC50(nM) = ~4.0 (IC50 = ~9.0 µM), Hill slope = 2.9. Kinetic values shown are ± SD.

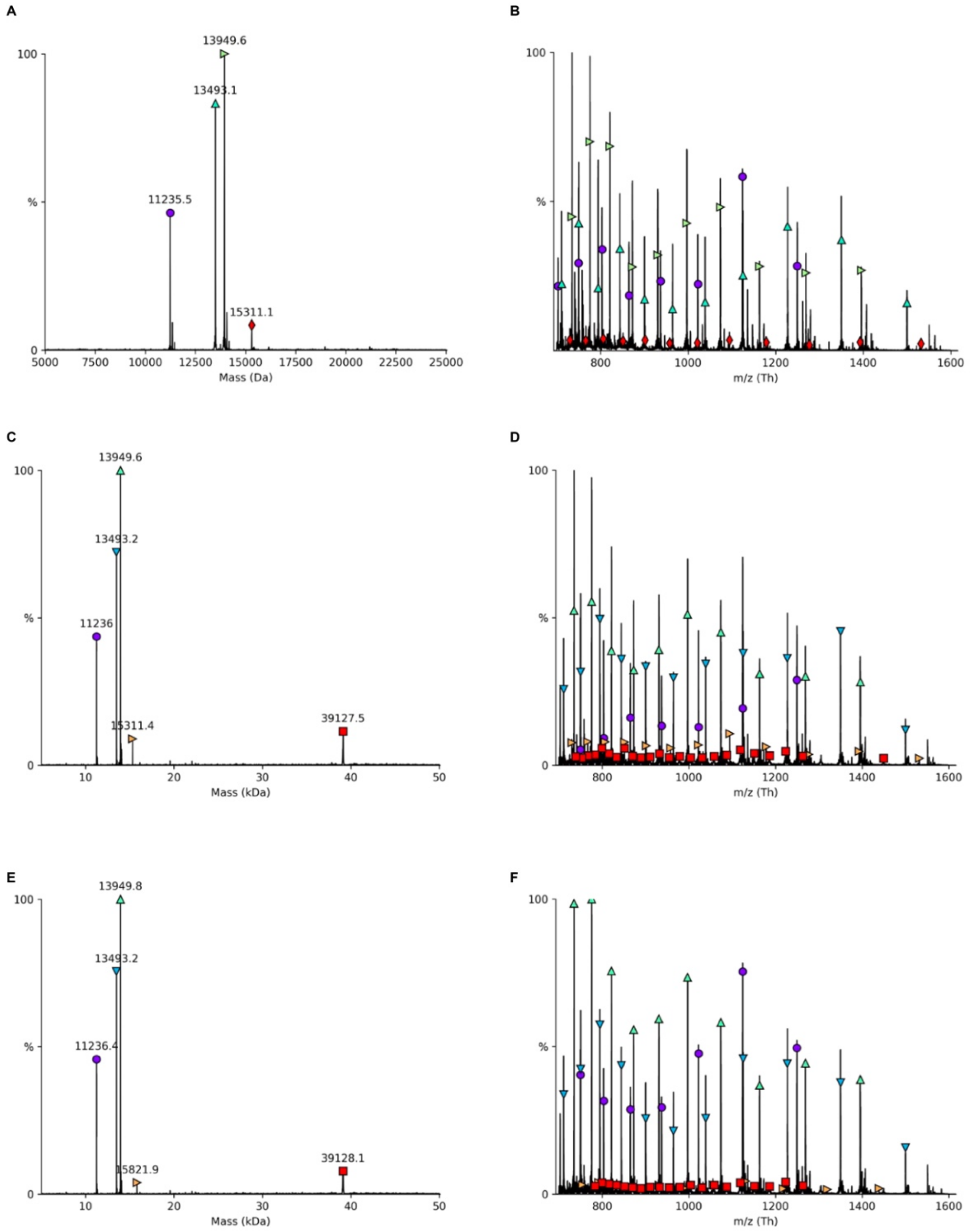

Figure S21. ESI analysis of 185 bp H3K27-N-methyl thiourea (MTU) nucleosome and NAD dependent reaction of crosslinking SIRT6 and H3K27-N-methyl thiourea nucleosome. (A) Deconvoluted mass spectrum of 185 bp H3K27MTU nucleosome: H4 (purple circle) calculated mass 11236.15 Da, found: 11235.5 Da; H2B (teal upward pointed triangle) calculated mass 13493.68 Da, found: 13493.1 Da; H2A (green rightward pointed triangle) calculated mass 13950.2 Da, found: 13949.6 Da; H3K27mtu (red diamond) calculated mass 15311.72 Da, found: 15311.1 Da. (B) Raw mass spectrum of 185 bp H3K27MTU nucleosome: H4 (purple circle); H2B (teal upward pointed triangle); H2A (green rightward pointed triangle); H3K27mtu (red diamond). (C) Deconvoluted mass spectrum of SIRT6 and H3K27MTU nucleosome following reaction without NAD: H4 (purple circle) calculated mass: 11236.15 Da, found: 11236.0 Da; H2B (teal downward pointed triangle) calculated mass: 13493.68 Da, found:

13493.2 Da; H2A (green upward pointed triangle) calculated mass: 13950.2 Da, found: 13949.6 Da; H3K27mtu (orange rightward pointed triangle) calculated mass: 15311.72 Da, found: 15311.4 Da; SIRT6 (red square) calculated mass: 39129.35 $[M+K]^+$, found: 39127.5 Da. (D) Raw mass spectrum of SIRT6 and H3K27MTU nucleosome following reaction without NAD: H4 (purple circle); H2B (teal downward pointed triangle); H2A (green upward pointed triangle); H3K27mtu (orange rightward pointed triangle); SIRT6 (red square). (E) Deconvoluted mass spectrum of SIRT6 and H3K27MTU nucleosome following reaction with NAD: H4 (purple circle) calculated mass 11236.15 Da, found: 11236.4 Da; H2B (teal downward pointed triangle) calculated mass: 13493.68 Da, found: 13493.2 Da; H2A (green upward pointed triangle) calculated mass: 13950.2 Da, found: 13949.8 Da; H3K27-NAD transition state analogue (orange rightward pointed triangle) calculated mass: 15821.97, found: 15821.9; SIRT6 (red square) calculated mass: 39129.35 $[M+K]^+$, found: 39128.1 Da. (F) Raw mass spectrum of SIRT6 and H3K27MTU nucleosome following reaction with NAD: H4 (purple circle); H2B (teal downward pointed triangle); H2A (green upward pointed triangle); H3K27-NAD intermediate analogue (orange rightward pointed triangle); SIRT6 (red square).

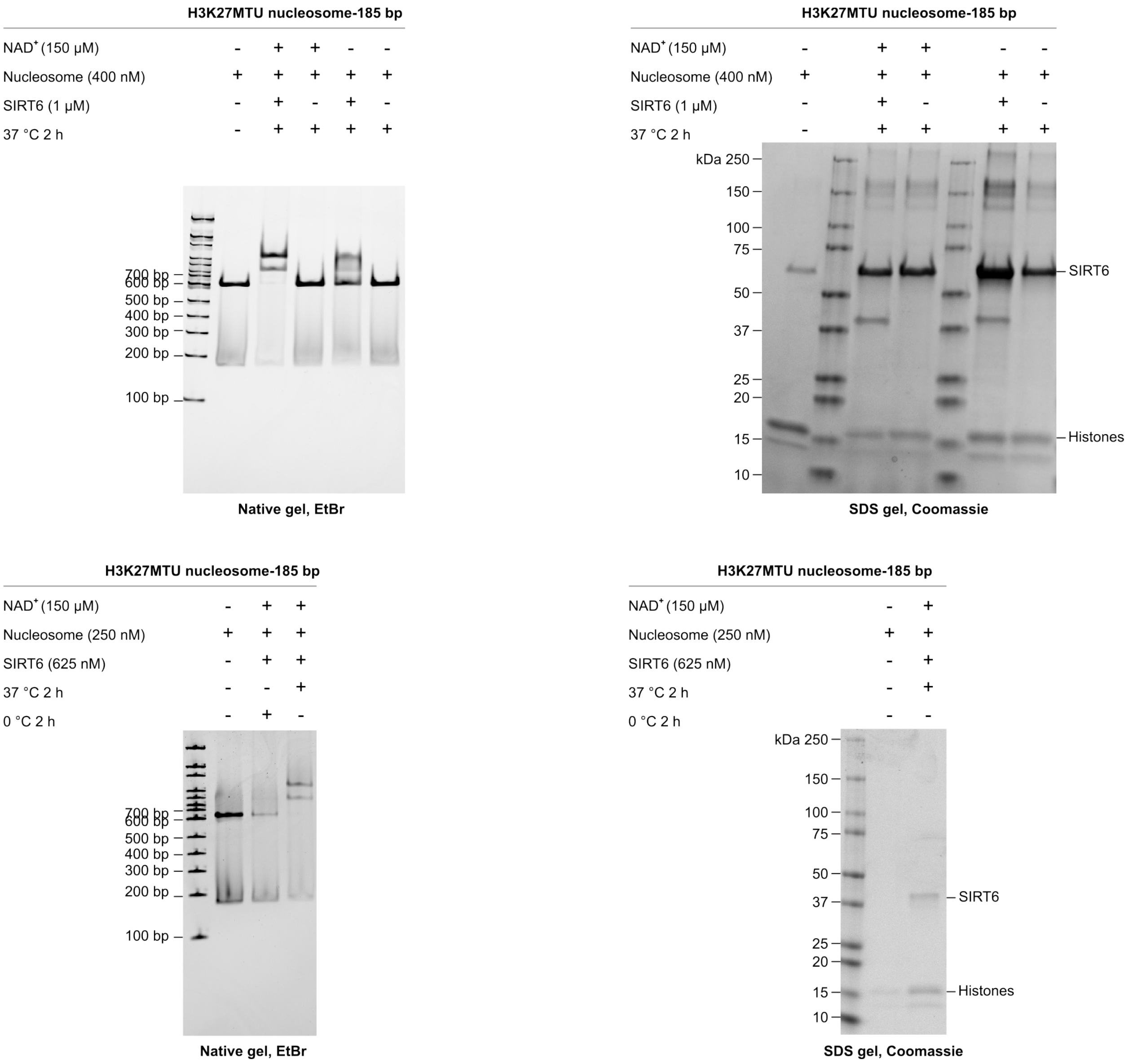

Figure S22. EMSA for NAD dependent reaction of crosslinking SIRT6 and H3K27MTU nucleosome. EMSA of WT SIRT6 binding with H3K27MTU nucleosome-185 bp at high concentration with or without NAD as (A) EtBr-stained native gel, and (B) Coomassie stained SDS PAGE; EMSA of WT SIRT6 binding with H3K27MTU nucleosome-185 bp at high concentration with or without NAD as (C) EtBr-stained native gel, and (D) Coomassie stained SDS PAGE.

Table S1: Cryo-EM data collection, refinement, and validation statistics

	SIRT6-nucloeosome (map A)<br>PDB nnnn<br>EMDB nnnnn
**Data collection and processing**	
Magnification	105,000
Voltage (kV)	300
Electron exposure (e^{-}/Å^2)	49.80
Defocus range (μm)	0.9-1.9
Pixel size (Å)	0.83
Symmetry imposed	C1
Initial particles images (no.)	10,305,397
Final particle images (no.)	124,274
Map resolution (Å)<br>FSC threshold	3.2<br>0.143
Map resolution range (Å)	3-6
**Refinement**	
Initial models used (PDB code)	3LZ0, 5Y2F, 8F86
Map sharpening *B* factor (Å^2)	-79.7
**Model composition**	
Non-hydrogen atoms<br>Protein residues<br>Nucleotides<br>Ligands	14070<br>1047<br>282<br>LIG: 1<br>ZN: 1
B factors (Å^2) Protein<br>Nucleotide<br>Ligand	76.29<br>117.17<br>155.41
**R.m.s. deviations**	
Bond lengths (Å)<br>Bond angles (°)	0.004 (0)<br>0.644 (3)
**Validation**	
MolProbity score<br>Clashscore<br>Poor rotamers (%)	1.68<br>4.55<br>0.00
**Ramachandran plot**	
Favored (%)<br>Allowed (%)<br>Disallowed (%)	96.88<br>2.83<br>0.29
**Model vs. Data**	
CC (mask)<br>CC (box)	0.77<br>0.76

Table S2: Input structural models and confidence

Domain/complex	Chain id	Input model	Level of confidence
Nucleosome	A-J	3LZ0	Atomic, side chains resolved
H2A	C,G	3LZ0	Atomic, side chains resolved
H2B	D,H	3LZ0	Atomic, side chains resolved
H3	A,E	3LZ0,5Y2F	Atomic, side chains resolved
H3 tail (residues 25-28)	A	8F86	Rigid Body docked
H4	B,F	3LZ0	Atomic, side chains resolved
DNA	I,J	3LZ0	Atomic, phosphate backbone resolved
SIRT6	K	5Y2F, AlphaFold2	Atomic, rigid body docked, AlphaFold2 modeled; partially side chains resolved or clear secondary structures resolved
SIRT6 (residues 2-16)	K	8F86/AlphaFold2	Rigid Body docked
SIRT6 (residues 17-83)	K	8F86/5Y2F	Rigid body docked
NAD analog	A	8F86	Rigid Body docked, density matches shape

Table S3. All the calculated V/[E] (Mean ± SD) for deacylation assays with H3K9 acylated nucleosome.

Acylation site and type	Sirt6 V/[E] (min-1)	Sirt6 replicate count (n)	Sirt1V/[E] (min-1)	Sirt1 replicate count (n)	Sirt2 V/[E] (min-1)	Sirt2 replicate count (n)	LHC V/[E] (min-1)	LHC replicate count (n)
H3K9ac	0.075±0.0063	8	<0.002	2	0.026±0.0021	2	0.021±0.0016	2
H3K9bu	0.044±0.0069	2	<0.002	2	0.013±0.00071	2	<0.002	2
H3K9cro	0.033±0.0030	2	<0.002	2	<0.002	2	0.0086±0.00055	2
H3K9hib	0.0013±0.0007	2	<0.002	2	<0.002	2	<0.002	2
H3K9succ	0.0044±0.00086	3	<0.002	2	<0.002	2	<0.002	2
H3K9lac	0.014±0.0012	2	<0.002	2	<0.002	2	<0.002	2
H3K9bhb	0.026±0.0025	2	<0.002	2	<0.002	2	<0.002	2

Acylation site and type	Sirt6 V/[E] (min-1)	Sirt6 replicate count (n)	Sirt6 G60A V/[E] (min-1)	Sirt6 G60A replicate count (n)	Sirt1V/[E] (min-1)	Sirt1 replicate count (n)	Sirt2 V/[E] (min-1)	Sirt2 replicate count (n)
H3K9ac	0.075±0.0063	8	0.02±0.0013	2	<0.002	2	0.026±0.0021	2
H3K9pr	0.041±0.0072	2	0.013±0.00062	2	<0.002	2	0.036±0.0031	2
H3K9bu	0.044±0.0069	2	0.013±0.0014	4	<0.002	2	0.013±0.00071	2
H3K9oct	0.46±0.088	2	0.40±0.062	2	<0.002	2	0.49±0.037	2
H3K9cro	0.033±0.0030	2	0.010±0.00088	2	<0.002	2	<0.002	2
H3K9hib	0.0013±0.00070	2	<0.002	2	<0.002	2	<0.002	2
H3K9succ	0.0044±0.00086	3	<0.002	2	<0.002	2	<0.002	2
H3K9lac	0.014±0.0012	2	<0.002	2	<0.002	2	<0.002	2
H3K9bhb	0.026±0.0025	2	0.0051±0.00067	2	<0.002	2	<0.002	2

Table S4. All the calculated V/[E] (Mean ± SD) for deacetylation assays with H3K9ac nucleosomes.

Acetylated Nucleosome	V/[E] (min-1)	replicate count (n)
**H3K9ac Nucleosome**	0.075±0.0063	8
**H3K4me3K9ac Nucleosome**	0.062±0.0033	2
**H3R8CitK9ac Nucleosome**	0.051±0.0099	3

Table S5. All the calculated V0/Vm (Mean ± SD) for activation/inhibition assays with H3K9ac nucleosome.

log[Myr(nM)]	V0/Vm	replicate count (n)
5	1.1±0.34	2
4	0.99±0.31	2
3	1.4±0.44	2
2	1.3±0.38	2
0	1.0±0.40	2

log[NAM(nM)]	V0/Vm	replicate count (n)
8	0.13±0.23	2
7	0.25±0.18	2
6.3	1.9±0.79	2
6	1.4±0.51	2
5	0.88±0.48	2
4	1.7±0.66	2
3	1.5±0.58	2
0	1±0.51	2

log[CL5D(nM)]	V0/Vm	replicate count (n)
4	0.14±0.09	2
3.7	0.65±0.12	2
3	0.70±0.20	2
2	1.2±0.21	2
0	1.0±0.10	2

log[MDL-800(nM)]	log[CL5D(nM)]	V0/Vm	replicate count (n)
5	4	0.42±0.11	2
5	3.7	0.84±0.20	2
5	0	1.0±0.18	2
